# Metabolomic and microarray analyses of adipose tissue of dapagliflozin-treated mice, and effects of 3-hydroxybutyrate on induction of adiponectin in adipocytes

**DOI:** 10.1038/s41598-018-27181-y

**Published:** 2018-06-11

**Authors:** Shigeki Nishitani, Atsunori Fukuhara, Jihoon Shin, Yosuke Okuno, Michio Otsuki, Iichiro Shimomura

**Affiliations:** 1 Departments of Metabolic Medicine, Osaka University Graduate School of Medicine, Suita, Osaka, Japan; 2 Departments of Adipose Management, Osaka University Graduate School of Medicine, Suita, Osaka, Japan; 3 Departments of Diabetes Care Medicine, Osaka University Graduate School of Medicine, Suita, Osaka, Japan

## Abstract

Sodium/glucose cotransporter 2 (SGLT2) inhibitor improves systemic glucose metabolism. To clarify the effect of dapagliflozin, we performed gene expression microarray and metabolomic analyses of murine adipose tissue. Three groups of mice were used; non-diabetic control KK mice (KK), diabetic KKAy mice (KKAy), and KKAy mice treated with dapagliflozin (KKAy + Dapa). Plasma glucose levels were significantly reduced in KKAy + Dapa compared with KKAy. Food consumption was larger in KKAy + Dapa than KKAy, and there were no significant differences in body and adipose tissue weight among the groups. Metabolomic analysis showed higher levels of many intermediate metabolites of the glycolytic pathway and TCA cycle in KKAy than KK, albeit insignificantly. Dapagliflozin partially improved accumulation of glycolytic intermediate metabolites, but not intermediate metabolites of the TCA cycle, compared with KKAy. Interestingly, dapagliflozin increased plasma and adipose 3-hydroxybutyric acid (3-HBA) levels. Microarray analysis showed that adipocytokines were downregulated in KKAy compared with KK mice, and upregulated by dapagliflozin. *In vitro*, 3-HBA induced β-hydroxybutyrylation of histone H3 at lysine 9 and upregulation of adiponectin in 3T3-L1 adipocytes independent of their acetylation or methylation. Our results suggest that 3-HBA seems to provide protection through epigenetic modifications of adiponectin gene in adipocytes.

## Introduction

Obesity is associated with metabolic disorders, such as diabetes mellitus, dyslipidemia, hypertension, and cardiovascular diseases^1^. Pathologically, obesity is also related to inflammation of the adipose tissue characterized by increased infiltration of immune cells, such as macrophages^2^. The adipose tissue is known to produce and secrete a variety of bioactive molecules known collectively as adipocytokines, such as adiponectin, leptin, IL-6, IL-1β, monocyte chemotactic protein-1 (MCP-1), and plasminogen activator inhibitor-1 (PAI-1)^3^. We reported previously that the mRNA expression levels of adiponectin were lower, whereas those of PAI-1 and TNF-α were higher in white adipose tissue (WAT) of obese diabetic KKAy mice compared with C57BL/6 J mice^4^.

Dapagliflozin is a selective competitive inhibitor of sodium/glucose cotransporter 2 (SGLT2), and has anti-diabetic effects through inhibition of renal glucose reabsorption^5^. Previous studies reported that the use of SGLT2 inhibitor reduced body weight^6^ and that treatment with empagliflozin resulted in at least 5% reduction in body weight^7^. Other studies, however, reported that obese diabetic KKAy mice treated with sergliflozin show suppression of body weight gains^8^, while treatment of db/db mice with remogliflozin resulted in body weight gain^9^.

Recent clinical trials demonstrated that treatment with SGLT2 inhibitor reduced cardiovascular events in patients with type 2 diabetes mellitus (T2DM). Furthermore, in the EMPA-REG OUTCOME clinical trial, treatment of T2DM patients with empagliflozin reduced the rate of primary composite of cardiovascular outcome and of death from any cause^10^. Another clinical trial, the CANVAS study, reported that canagliflozin reduced the risk of cardiovascular events in patients with T2DM^11^. Similarly, the CVD-REAL Study concluded that treatment with SGLT-2 inhibitors, such as canagliflozin, dapagliflozin, and empagliflozin, reduced the risk of cardiovascular death and hospitalization for heart failure^12^. The mechanism of cardiovascular risk reduction by SGLT2 inhibitor is not fully understood but includes improved glycemic control, reduction in body weight, blood pressure, and serum uric acid. In addition to these factors, it was reported recently that treatment with SGLT2 inhibitors was associated with high serum levels of 3-hydroxybutyric acid (3-HBA)^13^. 3-HBA is exclusively synthesized in the liver from acetyl-CoA through β-oxidation of fatty acids^14^. It is taken up in peripheral tissues by monocarboxylate transporter (MCT) and oxidized for ATP production^15^. 3-HBA is considered a signaling metabolite^16^. It is an endogenous inhibitor of histone deacetylases (HDACs), and suppresses oxidative stress by inducing the expression of MnSOD and catalase, with increased acetylation of lysine residues in histone H3^17^. Moreover, histone lysine residues are subject to direct modification by 3-HBA, which is associated with active gene expression^18^.

The present study was designed to clarify the effects of dapagliflozin in adipose tissues using gene expression microarray and metabolomic analyses in obese diabetic KKAy mice. The results showed that dapagliflozin increased both plasma and adipose tissue levels of 3-HBA, and importantly the induction of adiponectin expression by 3-HBA in 3T3-L1 adipocytes.

## Results

### Effects of dapagliflozin on KKAy mice

**Figure 1:**
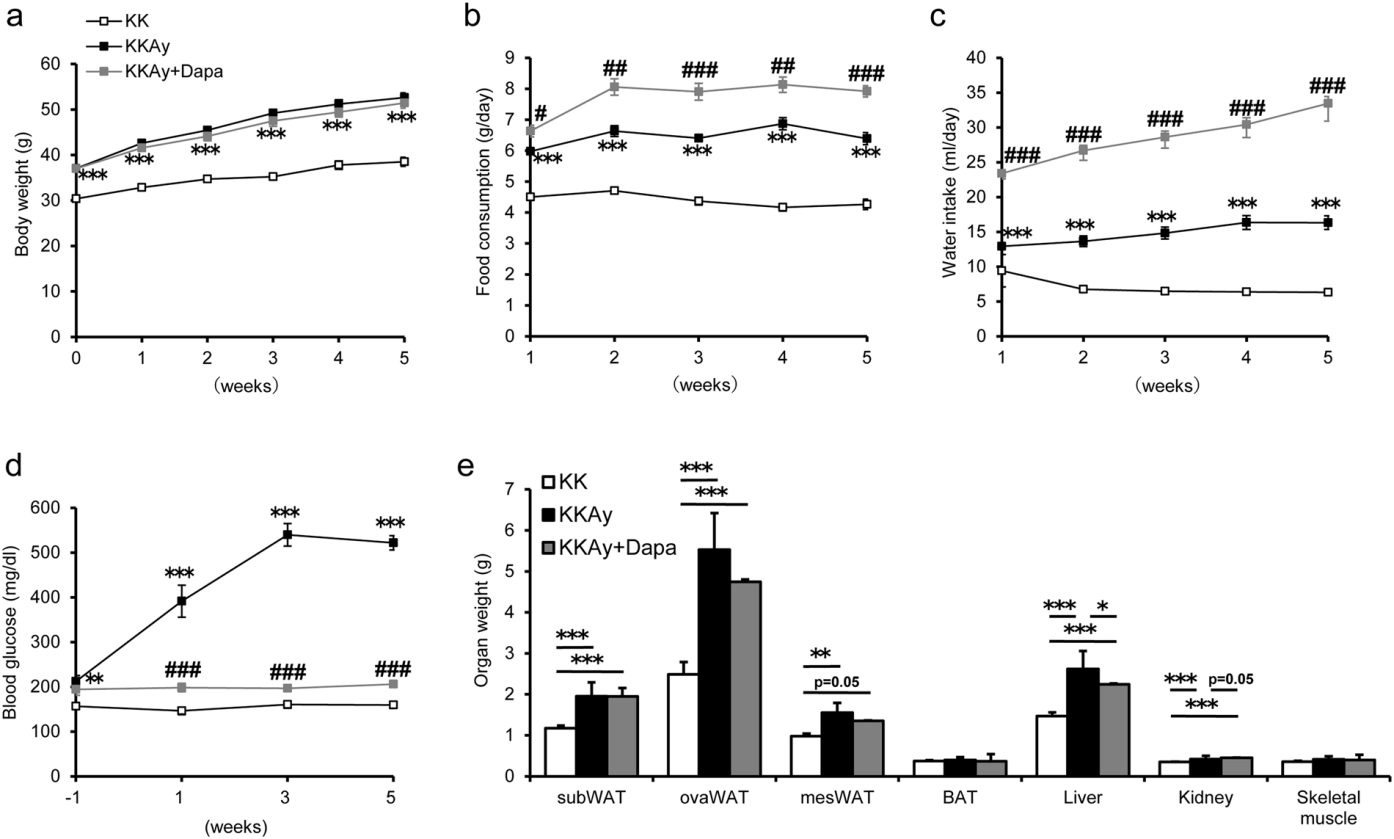
Changes in body weight, food consumption, water intake, blood glucose level, and organ weight of the three mice groups. Three groups of mice are used; non-diabetic control female KK mice (KK), diabetic female KKAy mice (KKAy), and female KKAy mice treated with dapagliflozin (KKAy + Dapa). (**a**) Body weight, (**b**) food consumption, and (**c**) water intake were measured weekly for 5 weeks. (**d**) Fasting blood glucose levels were measured every two weeks. Data are mean ± SEM (n = 6). **p < 0.01, ***p < 0.001, KK versus KKAy. #p < 0.05, ##p < 0.01, ###p < 0.001, KKAy versus KKAy + Dapa, by one-way ANOVA followed by post hoc analysis (Tukey-Kramer test). (**e**) Weight of subcutaneous WAT (subWAT), periovarian WAT (ovaWAT), mesenteric WAT (mesWAT), BAT, liver, kidney, skeletal muscle after 5 weeks of dapagliflozin treatment. Data are mean ± SEM (n = 6). *p < 0.05, **p < 0.01, ***p < 0.001, by one-way ANOVA followed by post hoc analysis (Tukey-Kramer test).

Three groups of mice were used; non-diabetic control female KK mice (KK), diabetic female KKAy mice (KKAy), and female KKAy mice treated with dapagliflozin (KKAy + Dapa) (n = 6, each). Dapagliflozin was prepared at 0.02 mg/ml concentration added to the drinking water. Based on the amount of water intake, the estimated dose of dapagliflozin was 13.0 ± 2.8 mg/kg body weight/day. Body weight was significantly larger in KKAy and KKAy + Dapa than KK mice during the study period (Fig. 1a), but there was no such difference between KKAy and KKAy + Dapa groups (Fig. 1a). KKAy mice carry heterozygous mutation of the agouti gene, resulting in obese diabetic phenotype due to highly increased appetite, associated with compromised hypothalamic control of appetite^19^. As expected, food and water intakes were significantly higher in both KKAy and KKAy + Dapa than KK (Fig. 1b,c), and this effect was observed on the first week of treatment with dapagliflozin (Fig. 1b,c). During the study period, plasma glucose levels were significantly higher in KKAy and KKAy + Dapa than KK (Fig. 1d). Treatment with dapagliflozin significantly reduced plasma glucose levels in KKAy + Dapa compared with KKAy (Fig. 1d). The weight of WAT (subcutaneous, periovarian, and mesenteric fat), was larger in KKAy and KKAy + Dapa than KK, but no such difference was noted between KKAy and KKAy + Dapa (Fig. 1e). Brown adipose tissue (BAT) weight was not significantly different among the three groups (Fig. 1e). Liver weight was significantly larger in KKAy and KKAy + Dapa than KK, and was significantly lower in KKAy + Dapa than KKAy (Fig. 1e). Kidney weight was significantly larger in KKAy and KKAy + Dapa than KK, and tended to be larger in KKAy + Dapa than KKAy (Fig. 1e). Skeletal muscle weight was similar in the three groups (Fig. 1e).

### Effects of dapagliflozin on blood parameters

**Figure 2:**
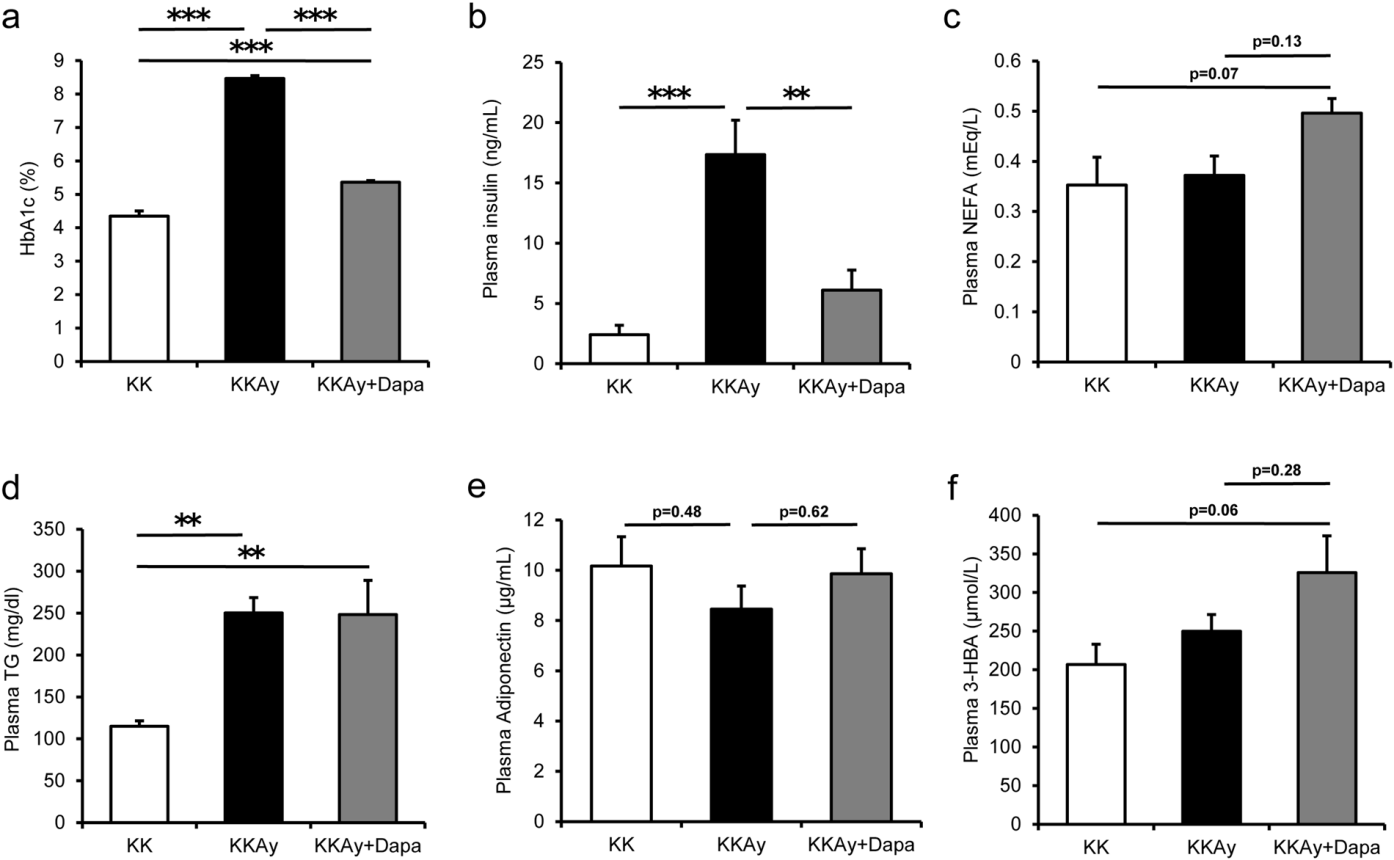
Plasma levels of glycated HbA1c, insulin, NEFA, TG, adiponectin, and 3-HBA. (**a**) Glycated HbA1c (HbA1c), (**b**) plasma insulin, (**c**) plasma NEFA, (**d**) plasma TG, (**e**) plasma adiponectin, and (**f**) plasma 3-HBA levels were measured after 5 weeks of treatment with dapagliflozin. Data are mean ± SEM (n = 6). *p < 0.05, **p < 0.01, ***p < 0.001, by one-way ANOVA followed by post hoc analysis (Tukey-Kramer test).

Glycated hemoglobin levels were significantly higher in KKAy than KK, and significantly lower in KKAy + Dapa than KKAy (Fig. 2a), indicating the strong glucose-lowering effect of dapagliflozin. Plasma insulin levels were significantly higher in KKAy than KK, and significantly lower in KKAy + Dapa than KKAy (Fig. 2b), indicating improvement in insulin resistance. Plasma triglyceride levels were also higher in both KKAy and KKAy + Dapa relative to KK. The levels of plasma non-esterified fatty acids (NEFA) was 0.38 ± 0.06 mEq/L in KKAy before treatment, and 0.37 ± 0.04 mEq/L in KKAy and 0.50 ± 0.03 mEq/L in KKAy + Dapa at the end of treatment. NEFA levels tended to be higher in KKAy + Dapa than KK and KKAy (Fig. 2c,d). Plasma adiponectin levels also tended to be lower in KKAy than KK, and higher in KKAy + Dapa than KKAy, albeit insignificantly (Fig. 2e). Plasma levels of 3-HBA, a ketone body, were 215.3 ± 92.7 μmol/L in KKAy before treatment, and 249.7 ± 21.7 μmol/L in KKAy and 325.8 ± 47.4 μmol/L in KKAy + Dapa at the end of treatment. 3-HBA levels tended to be higher in both KKAy and KKAy + Dapa than KK (Fig. 2f). Plasma levels of corticosterone showed no significant differences among groups (Supplementary Fig. S1a). Plasma levels of leptin were significantly higher in KKAy than KK, and lower in KKAy + Dapa than KKAy (Supplementary Fig. S1b).

### Metabolomic analysis of adipose tissue

**Figure 3:**
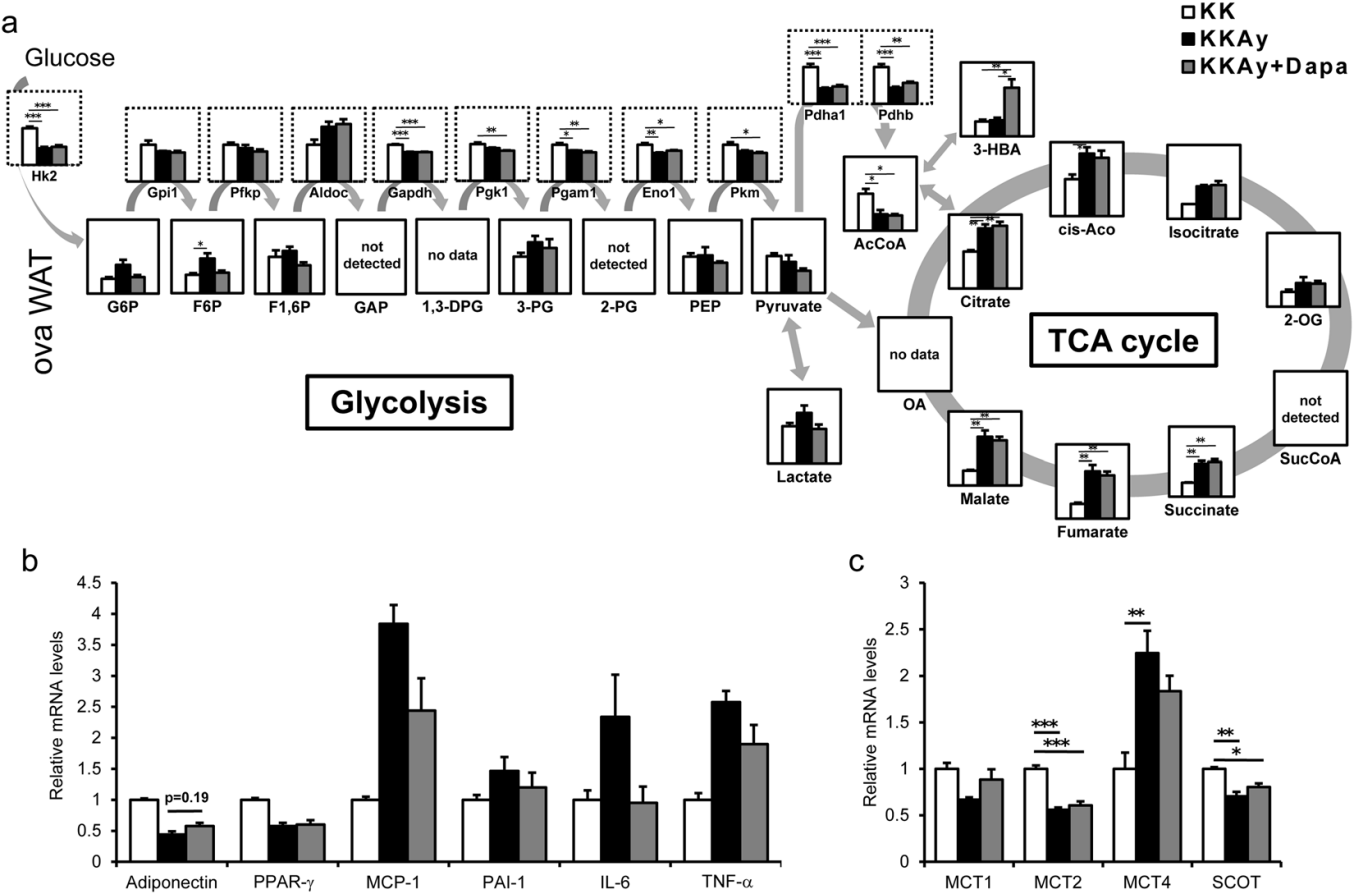
Results of metabolomic and microarray analyses of periovarian WAT (ovaWAT). All analysis were performed after 5 weeks of dapagliflozin treatment. (**a**) Relative levels of metabolites and gene expressions associated with glycolytic and tricarboxylic cycle (TCA cycle) pathways. Relative levels of metabolites and gene expressions were surrounded by solid line and bottled line, respectively. (**b**,**c**) Relative expression levels of genes associated with adipocytokines (**b**) and the metabolism of ketone body (**c**). Data are normalized to the values of metabolites or gene expression levels of KK mice, and expressed as mean ± SEM (n = 4). *p < 0.05, **p < 0.01, ***p < 0.001, by one-way ANOVA followed by post hoc analysis (Tukey-Kramer test). G6P, glucose 6-phosphate; F6P, fructose 6-phosphate; F1,6P, fructose 1,6-diphosphate; GAP, glyceraldehyde 3-phosphate; 1,3-DPG, 1,3-di phosphoglycerate; 3-PG, 3-phosphoglycerate; 2-PG, 2-phosphoglycerate; PEP, phosphoenolpyruvate; AcCoA, acetyl CoA; OA, oxaloacetate; cis-Aco, cis-aconitate; 2-OG, 2-oxoglutarate; SucCoA, succinyl-CoA; 3-HBA, 3-hydroxybutyrate; Hk2, hexokinase 2; Gpi1, glucose phosphate isomerase 1; Pfkp, phosphofructokinase, platelet; Aldoc, aldolase C; Gapdh, glyceraldehyde-3-phosphate dehydrogenase; Pgk1, phosphoglycerate kinase-1; Pgam1, phosphoglycerate mutase 1; Eno1, enolase 1; Pkm, pyruvate kinase, muscle; Pdha1, pyruvate dehydrogenase E1 alpha 1; Pdhb, pyruvate dehydrogenase (lipoamide) beta; PPAR-γ, peroxisome proliferator activated receptor gamma; MCP-1, Monocyte chemoattractant protein-1; PAI-1, plasminogen activator inhibitor-1; IL-6, interleukin 6; TNF-α, tumor necrosis factor alpha; MCT1, monocarboxylic acid transporters 1; MCT2, monocarboxylic acid transporters 2; MCT4, monocarboxylic acid transporters 4; SCOT, succinyl-CoA-3-oxaloacid CoA transferase.

To investigate the effects of dapagliflozin in adipose tissue, we analyzed the metabolites in periovarian WAT using CE-MS analysis. The levels of metabolites of the glycolytic pathway and tricarboxylic acid (TCA) cycle tended to be higher in KKAy than KK. The levels of metabolites of the glycolytic pathway tended to be lower in KKAy + Dapa than KKAy while those of metabolites of the TCA cycle were not different between these two groups (Fig. 3a). 3-HBA level was significantly higher in KKAy + Dapa than KK and KKAy (Fig. 3a).

Carnitine plays an essential role in the transfer of fatty acids across the inner mitochondrial membrane. In the first step of transport, fatty acyl CoA is converted to acylcarnitine, which can enter the mitochondria for energy production via β-oxidation^20^. Palmitoylcarnitine, and acylcarnitine (16:2), (18:0), (18:1), (18:2), and (20:1) were elevated in KKAy + Dapa compared with KK or KKAy (Supplementary Fig. S2), suggesting enhanced mitochondrial oxidation of fatty acids by dapagliflozin treatment.

### Microarray analysis of adipose tissue

We also performed microarray analysis of gene expression in periovarian WAT. The expression levels of pdha1 and pdhb, catalyzing the conversion of pyruvate into acetyl-CoA, showed a significant 58% and 56% reduction in KKAy than KK (Fig. 3a). In addition, expression levels of gapdh, pgam1, and eno1 were significantly reduced in KKAy compared with KK (Fig. 3a). Hk2 expression levels were also significantly reduced in KKAy compared with KK (Fig. 3a). Next, we measured pyruvate carboxylase (PCX) activity in periovarian WAT of KK, KKAy and KKAy + Dapa. PCX catalyzes the physiologically irreversible carboxylation of pyruvate to form oxaloacetate (OAA), and plays an anaplerotic role to supply intermediate metabolites of the TCA cycle for biosynthesis of amino acids and fatty acids^21^. PCX activity was significantly higher in periovarian WAT of KKAy + Dapa than that of KK or KKAy (Supplementary Fig. S3).

Expressions of genes involved in lipogenesis, such as acly, acaca, fasn elovl6, and scd1, were significantly lower in KKAy and KKAy + Dapa than KK (Supplementary Fig. S4a). These expressions tended to be higher in KKAy + Dapa than KKAy (Supplementary Fig. S4a). In addition, expressions of genes involved in lipolysis, such as lipe, pnpla2, and mgll were significantly reduced in KKAy and KKAy + Dapa than KK, and showed tendency to elevate in KKAy + Dapa compared with KKAy (Supplementary Fig. S5).

With regard to adipocytokines, adiponectin gene expression tended to be lower in KKAy than KK, and to be higher in KKAy + Dapa than KKAy (Fig. 3b). In contrast, the expression levels of inflammation-related genes, such as MCP-1, PAI-1, IL-6, TNF-α, tended to be higher in KKAy than KK, and lower in KKAy + Dapa than KKAy (Fig. 3b). With regard to insulin signaling molecules, insr (insulin receptor), irs1 (IRS-1), and slc2a4 (Glut4) expressions were significantly lower in KKAy than KK and tended to be higher in KKAy + Dapa than KKAy (Supplementary Fig. S6).

MCT1 and MCT2 are monocarboxylate transporters that import 3-HBA into peripheral tissues, while MTC4 exports it^15^. Succinyl-CoA-3-oxaloacid CoA transferase (SCOT) is the major enzyme in extrahepatic ketone body catabolism^22^. The mRNA expression levels of MCT1, MCT2, and SCOT, tended to be lower in KKAy than KK, and higher in KKAy + Dapa than KKAy (Fig. 3c). Furthermore, the mRNA expression level of MCT4 tended to be higher in KKAy than KK, and lower in KKAy + Dapa than KKAy (Fig. 3c). Next, we measured SCOT activity in periovarian WAT of KK, KKAy and KKAy + Dapa. SCOT activity was significantly higher in KKAy + Dapa than KKAy (Supplementary Fig. S7).

### Metabolomic analysis and microarray analysis of liver

To investigate the effects of dapagliflozin in liver, we analyzed the metabolites in liver using CE-MS analysis. The levels of phosphoenolpyruvic acid (PEP) and 3-HBA were significantly higher in KKAy + Dapa than KKAy (Supplementary Fig. S8). Ketogenesis is limited to hepatocytes due to restricted expression of the ketogenic enzyme mitochondrial 3-hydroxymethylglutaryl-CoA synthase (HMGCS2)^23^. In liver, AcCoA levels tended to be lower in KKAy + Dapa compared with KK or KKAy, suggesting the synthesis of 3-HBA from AcCoA (Supplementary Fig. S8). Hepatic substrates for gluconeogenesis, such as alanine, lactate, glycerol-3-phosphate (G3P), and dihydroxyacetone phosphate (DHAP) tended to be lower in KKAy + Dapa than KK and KKAy (Supplementary Fig. S9a).

We also performed microarray analysis of gene expression in liver. The expression levels of genes of the reaction enzymes of the glycolytic pathway, such as glucose phosphate isomerase 1 (Gpi1), glyceraldehyde 3-phosphate dehydrogenase (Gapdh), and enolase 1 (Eno1) were significantly lower in KKAy + Dapa than KKAy (Supplementary Fig. S8).

Expression levels of phosphoenolpyruvate carboxykinase-1 (Pck1) were significantly higher in KKAy + Dapa than KK or KKAy (Supplementary Fig. S9b), and g6pc tended to increase in KKAy + Dapa compared with KKAy (Supplementary Fig. S9b), suggesting the upregulation of gluconeogenesis by dapagliflozin treatment. Expressions of genes involved in lipogenesis in liver, such as acaca and elovl6, were significantly higher in KKAy than KK (Supplementary Fig. S4b). Expressions of acaca, fasn, elovl6, and scd1 were significantly lower in KKAy + Dapa than KKAy (Supplementary Fig. S4b). Zinc alpha 2-glycoprotein (Azgp1) is a lipid mobilizing factor^24^, and its expressions were significantly higher in KKAy + Dapa than KK or KKAy (Supplementary Fig. S4c).

### Effects of 3-hydroxybutyric acid on mRNA expression levels of adiponectin and intracellular adiponectin protein levels in 3T3-L1 adipocytes

**Figure 4:**
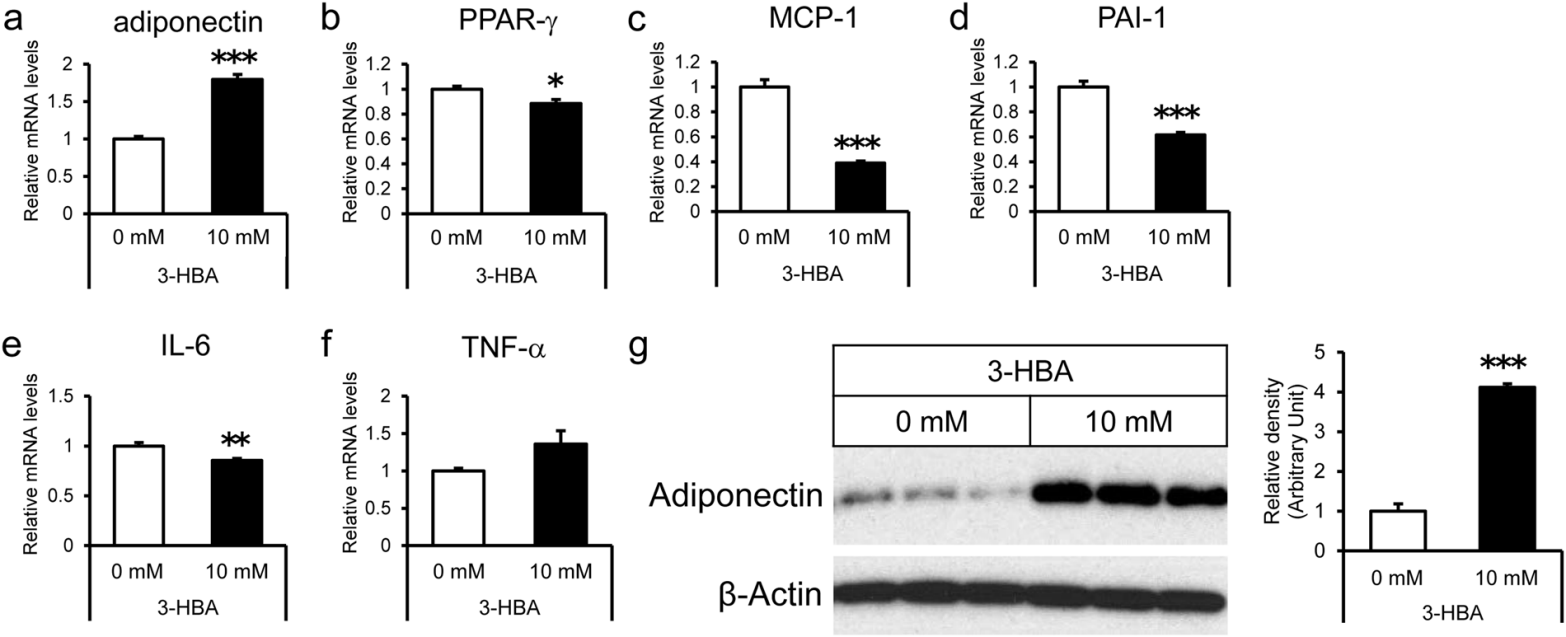
Effects of 3-HBA on mRNA expression levels and intracellular protein levels in 3T3-L1 adipocytes. On day 7 after differentiation, the medium of 3T3-L1 cells were replaced with KRBB supplemented with 0 or 10 mM 3-HBA and incubated for 24 hour. (**a**,**f**) The relative mRNA expression levels of adiponectin (**a**), PPAR-γ (**b**), MCP-1 (**c**), PAI-1 (**d**), IL-6 (**e**), and TNF-α (**f**) in 3T3-L1 adipocytes were measured by quantitative real-time PCR. Data are normalized to the level of 36B4 mRNA, and expressed as mean ± SEM (n = 6). (**g**) Intracellular protein levels of adiponectin and β-Actin in 3T3-L1 adipocytes using western blot analysis. *Left panel;* Representative western blot analysis. *Right panel;* Quantitative analysis of adiponectin contents in the left panel. Data are mean ± SEM (n = 3). *p < 0.05, **p < 0.01, ***p < 0.001.

3-HBA provides protection against apoptosis of cardiomyocytes^25^. Based on this observation, we postulated that 3-HBA also provides protection in adipocytes. To test the latter, we incubated 3T3-L1 adipocytes in the presence or absence of 3-HBA. The presence of 3-HBA was associated with overexpression of adiponectin mRNA and downregulation of inflammation-related genes, such as MCP-1, PAI-1, and IL-6 (Fig. 4a–f, n = 6). Western blot analysis confirmed that the intracellular protein levels of adiponectin were significantly higher in 3T3-L1 adipocytes incubated with 3-HBA than without 3-HBA (Fig. 4g, n = 3). Treatment with 3-HBA did not affect glycerol release from 3T3-L1 adipocytes (Supplementary Fig. S10), suggesting no major effect of 3-HBA on lipolysis.

### Mechanism of 3-hydroxybutyric acid-induced increase in adiponectin

**Figure 5:**
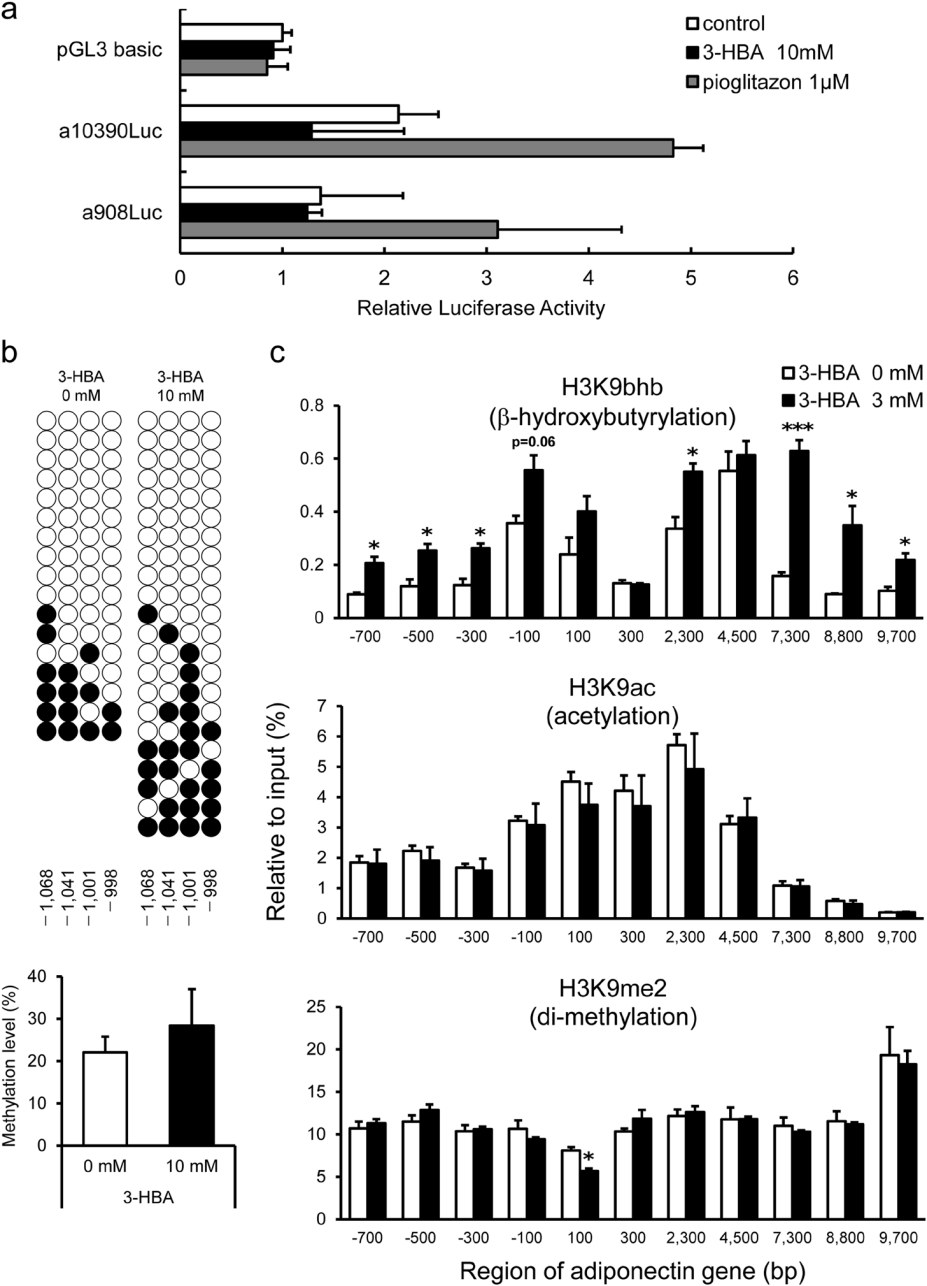
Effects of 3-HBA on adiponectin gene in 3T3-L1 adipocytes. (**a**) Promoter analysis of adiponectin gene in 3T3-L1 adipocytes. A series of fragments of the 5′-flanking region of the human adiponectin gene were subcloned upstream of the luciferase reporter gene as described in Materials and Methods. On day 7 after differentiation, each promoter/reporter construct was transfected into 3T3-L1 adipocytes, and next day, the media of 3T3-L1 cells were replaced with KRBB supplemented with or without 10 mM 3-HBAs or 1 μM pioglitazone. Luciferase activity was measured after 24-hr incubation. Luciferase values were normalized by an internal CMV-Renilla control and expressed as relative luciferase activity. Data are mean ± SEM (n = 3). (**b**) Adiponectin promoter region bisulfite sequencing analysis of 3T3-L1 adipocytes. On day 7 after differentiation, the media of 3T3-L1 cells were replaced with KRBB supplemented with 0 or 10 mM 3-HBA and incubated for 24 hr. *Top panel;* Each circle represents sequencing results of independent clones. Open circles: unmethylated CpGs, solid circles: methylated CpG. The CpG position relative to upstream transcription start site of mouse adiponectin gene is shown below each column. *Bottom panel;* Percentage of 5-methylcytosine. Data are mean ± SEM of three independent samples (n = 3). (**c**) ChIP-qPCR analysis of histone H3 tail at lysine 9 modifications on the adiponectin gene in 3T3-L1 adipocytes. On day 7 after differentiation, the media of 3T3-L1 cells were replaced with KRBB supplemented with 0 or 3 mM 3-HBA and incubated for 24 hr. The genomic DNA was precipitated by antibodies against β-hydroxybutyrylated histone H3 at lysine 9 (H3K9bhb), acetylated histone H3 at lysine 9 (H3K9ac), di-methylated histone H3 at lysine 9 (H3K9me2). ChIP signals of each region of adiponectin gene were detected by quantitative real-time PCR and normalized to input signal as relative to input (%). Data are mean ± SEM (n = 3). *p < 0.05, **p < 0.01, ***p < 0.001.

To determine the mechanism of 3-HBA-induced increase in adiponectin expression in 3T3-L1 adipocytes, promoter analysis was performed as described in detail previously^26,27^. Luciferase activity was induced with pioglitazone in a10390Luc or a908Luc containing 10390 bp or 908 bp of human adiponectin promoter, respectively^27^, but not with 3-HBA (Fig. 5a, n = 3). Based on these results, we speculated that 3-HBA-related induction of adiponectin gene is mediated through epigenetic regulation. As reported by Kim *et al*.^28^, obesity-induced pro-inflammatory cytokines promote DNA methylation of adiponectin promoter, resulting in reduction of adiponectin expression. We conducted bisulfite sequencing analysis of 3T3-L1 adipocytes treated with or without 3-HBA. 3-HBA had no effect on the DNA methylation levels of the adiponectin gene (Fig. 5b). With regard to other epigenetic modifications, previous studies reported that adiponectin gene expression is associated with methylation or acetylation of histone H3 at lysine 9 (H3K9) in 3T3-L1 adipocytes^29^ while another study described lysine β-hydroxybutyrylation (Kbhb), a new type of histone modification^18^. To assess modification of the histone H3K9, we performed chromatin immunoprecipitation assay (ChIP assay) followed by qPCR analysis for adiponectin gene in 3T3-L1 adipocytes (Fig. 5c). 3-HBA induced significant upregulation of Kbhb modification of H3K9 upstream (−700 bp to −100 bp) and downstreatm ( +2300 bp and +7300 bp to +9700 bp) of the translation initiation site of adiponectin gene, whereas acetylation or di-methylation did not (Fig. 5c). These results indicate that induction of adiponectin expression by 3-HBA is associated with enhanced modification of H3K9 by Kbhb in adiponectin gene.

## Discussion

Increased food intake was observed in SGLT2-deficient mice^30^, and in diet-induced obese rats treated with dapagliflozin^31^. KKAy mouse is a hyperphagic obese diabetic model due to the antagonism of hypothalamic melanocortin receptor-4 by ectopic expression of the agouti protein^32,33^. In the present study, food intake was significantly higher in KKAy + Dapa than KKAy. Leptin is a satiety hormone that reduces appetite^34^, and we found that plasma leptin levels were significantly lower in KKAy + Dapa than KKAy in the current study. Regulation of appetite involves a balance between excitatory and inhibitory processes. Agouti gene mutation stimulates, whereas, leptin reduces appetite by opposing effects on paraventricular nucleus (PVN) of hypothalamus (Supplementary Fig. S11). Reduced plasma leptin levels by dapagliflozin is supposed to fail in suppression of appetite resulting in further enhancement of hyperphagia. Therefore, reduced leptin levels might be partially responsible for the hyperphagia at least in KKAy + Dapa.

There was no significant difference in the weight of WAT between KKAy and KKAy + Dapa. In periovarian WAT, expressions of genes involved in lipolysis, such as lipe, pnpla2, and mgll showed tendency to elevate, and acylcarnitines tended to be higher in KKAy + Dapa compared with KK or KKAy, suggesting enhancement of both lipolysis and mitochondrial oxidation of fatty acids by dapagliflozin treatment. On the other hand, expressions of genes associated with lipogenesis tended to be higher in periovarian WAT of KKAy + Dapa than KKAy. Collectively, dapagliflozin-induced lipolysis and fatty acid oxidation should be partially compensated by modest increase of lipogenesis in periovarian WAT, resulting in no significant changes in fat weight in the current study. In another condition of enhanced lipolysis by chronic β3-adrenergic receptor stimulation, Mottillo reported the coupling of lipolysis, fatty acid oxidation, and lipogenesis in adipocytes^35^. When lipolysis is activated, greater flux of fatty acids into mitochondria activates fatty acid oxidation. In addition, lipolysis-dependent generation of ligands for PPARs upregulate transcription of lipogenic enzymes^35^. In adipocyte-specific ATGL-deficient mice, a model of reduced adipocyte lipolysis, fatty acid oxidation and lipogenesis were also impaired^36^. Treatment with dapagliflozin exhibited similar metabolic changes in adipose tissues with these lipolysis-modified models.

Previous report showed that expressions of lipogenic genes in liver of HFD-induced obese diabetic models were reduced by the treatment with tofogliflozin or empagliflozin^37,38^. In these reports, both body weight and liver weight were reduced in pair-feeding conditions against control mice. In another report, expressions of lipogenic genes in liver of amylin NASH models were reduced by the treatment with ipragliflozin without changes in body weight^39^. In the current study, dapagliflozin did not reduce body weight probably because of excess calorie intake by hyperphagia. In this condition, liver weight was significantly decreased in KKAy + Dapa compared with KKAy. Moreover, we revealed for the first time that expressions of lipogenic genes were significantly reduced in KKAy mice treated with dapagliflozin without pair-feeding. There are two explanations for the reduced lipogenesis in liver by dapagliflozin. The first is the direct effect of dapagliflozin against hepatocytes. In palmitic acid-induced HepG2 cells, treatment with dapagliflozin significantly attenuates the mRNA expression of fasn and acaca^40^. The second is the effect of lipid mobilizing factor, Azgp1 (zinc alpha 2-glycoprotein). Injection of Azgp1 expression plasmid reduces hepatic TG content and expressions of lipogenic genes^24^. In the current study, expressions of Azgp1 were significantly elevated in liver of KKAy + Dapa compared with KKAy.

It was reported that tofogliflozin, another SGLT2 inhibitor, increases plasma adiponectin levels and reduces body weight in KKAy mice^41^. In our models, dapagliflozin tended to increase plasma adiponectin levels and upregulate adiponectin expression in adipose tissues, compared with KKAy albeit insignificantly, and did not induce significant reduction in body weight compared with KKAy, probably due to hyperphagia. Overfeeding enhances total intake of fat and amino acids (components of normal chow), and excess lipids or glutamate results in reduced adiponectin secretion^42,43^. Collectively, in our setup, overfeeding might have masked the effects of dapagliflozin on the enhancement of adiponectin expression.

The present results are similar to those of Nagao *et al*.^43^ who conducted metabolomic analysis of epididymal adipose tissue of C57BL/6 J and ob/ob mice, and showed that many metabolites of the TCA cycle, such as 2-oxoglutarate, succinate, fumarate and malate, were significantly higher in ob/ob mice than in C57BL/6J mice. With respect to the glycolytic pathway in periovarian WAT, amount of intermediate metabolites, such as G6P, F6P, and F1,6P tended to be higher in KKAy than KK. Microarray analysis indicated that expressions of insulin signaling molecules, including insr, irs1, and slc2a4 (Glut4), were significantly lower in KKAy than KK, and expression levels of gapdh, pgam1, Eno1, pdha1 and pdhb were significantly lower in KKAy than KK. Considered together, reduced expression of these distal glycolytic enzymes might be responsible for the accumulation of intermediate metabolites of glycolytic pathway in periovarian WAT of KKAy. In KKAy + Dapa, amount of these intermediate metabolites were normalized to the levels of KK. There were no differences in expressions of gapdh, pgam1, Eno1, pdha1 and pdhb between KKAy and KKAy + Dapa, however, PCX activity was significantly higher in periovarian WAT of KKAy + Dapa than that of KK, suggesting the increase of outflow from pyruvate to oxaloacetate. Upregulation of PCX activity might be partially responsible for the improvement of glycolytic pathway by dapagliflozin.

In this study, we first found that 3-HBA upregulated the mRNA expression level of adiponectin and intracellular adiponectin protein level in 3T3-L1 adipocytes. Adiponectin expression is regulated by both epigenetic modifications^28,29^ and promoter activity^26,27^. We identified previously a functional PPAR-responsive element and a distal enhancer in adiponectin gene^26,27^. Other studies showed that obesity-induced pro-inflammatory cytokines promoted DNA hypermethylation of a particular region of the adiponectin promoter, and suppressed adiponectin expression through epigenetic regulation in 3T3-L1 adipocytes^28^. Furthermore, acetylation and methylation of histone H3K9 in the adiponectin gene were reported to be associated with adiponectin expression in 3T3-L1 adipocytes^29^. In the present study, while 3-HBA induced adiponectin expression in 3T3-L1 adipocyte, changes were neither noted in adiponectin promoter activity, DNA methylation in adiponectin promoter, nor acetylation or methylation of histone H3K9 on adiponectin gene. We focused on a new type of histone modification, lysine β-hydroxybutyrylation^18^. During prolonged fasting, histone is subject to modification by 3-HBA, which is an active gene mark^18^. In the present study, treatment with 3-HBA induced both adiponectin expression and H3K9bhb in adiponectin gene, independent of their acetylation or methylation. Considering that adiponectin has anti-inflammatory and anti-atherogenic properties^44^, 3-HBA might have protective roles through epigenetic modifications of adiponectin gene. Further study is required to reveal the existence of H3K9bhb in adipose tissues of diabetic patients treated with SGLT2 inhibitor.

In the present study, we identified the effect of dapagliflozin in KKAy mice. We suppose working hypothesis as follows (Supplementary Fig. S11). Ectopic expression of agouti protein in KKAy mice stimulates appetite^32,33^. Excess calorie intake due to hyperphagia results in reduction of adiponectin expression, induction of inflammatory cytokines, and inhibition of lipogenesis, lipolysis, and fatty acid oxidations in periovarian WAT. Treatment with dapagliflozin reduces plasma leptin levels, enhances further hyperphagia, whereas, it improves dysregulation of adipocytokines and lipid metabolism in periovarian WAT.

In summary, dapagliflozin improved glucose levels and induced 3-HBA in obese diabetic mice. 3-HBA can enhance adiponectin expression in adipocytes through direct modification of histone H3K9. Identification of the target genes of 3-HBA is important to understand the molecular mechanisms of SGLT2 inhibitors in protection against cardiovascular events.

## Methods

### Animal studies

6-week-old female KK mice and KKAy mice were obtained from CLEA Japan (Tokyo, Japan) and acclimated for 2 week before the experiment. Mice were housed individually in sterile cages, maintained in a room under controlled temperature (23 ± 1.5 °C) and humidity (45 ± 15%) on a 12-h dark/12-h light cycle, and had free access to water and chow (MF; Oriental Yeast, Tokyo). Dapagliflozin (Cay-11574) was purchased from Cayman Chemical (Ann Arbor, MI). Dapagliflozin was dissolved in 100% ethanol at 125 mg /ml, followed by dilution with distilled water (160 μl of dapagliflozin mixture/1000 ml water, with final concentration of 0.02 mg/ml dapagliflozin). This concentration of ethanol (0.016% ethanol) did not affect amount of water intake, body weight and plasma 3-HBA concentrations (data not shown). Drinking water bottles were changed twice a week. Dapagliflozin treatment was conducted by adding 0.02 mg/ml dapagliflozin in drinking water. Three groups of mice were used; non-diabetic control female KK mice (KK), diabetic female KKAy mice (KKAy), and female KKAy mice treated with dapagliflozin (KKAy + Dapa). Food consumption was determined by weighing the metal cage top, including the food. Water intake was determined by measuring the fluid in drinking water bottles. Five weeks after the start of dapagliflozin treatment, the animal was fasted for 4 hours then anesthetized with isoflurane and blood samples were collected from the inferior vena cava, before sacrifice. Samples from seven tissues were collected, including subcutaneous fat, periovarian fat, mesenteric fat, BAT, liver, kidney, and skeletal muscle (gastrocnemius muscle). Gene expression microarray and metabolomic analyses were performed to compare the effects of dapagliflozin in periovarian adipose tissues. All mice studies were approved by the Ethics Review Committee for Animal Experimentation of Osaka University, Graduate School of Medicine, and carried out in accordance with the Institutional Animal Care and Use Committee Guidelines of Osaka University.

### Measurements of blood parameters

Blood glucose levels were measured by tail vein sampling every 2 weeks from 1 week before the start of dapagliflozin treatment using a portable glucose meter (Glutest Neo alpha, Sanwa Kagaku Kenkyusho, Nagoya, Japan). HbA1c was measured by tail vein sampling at 5 weeks after the start of dapagliflozin using the DCA 2000 HbA1c (Siemens, Tokyo). Plasma concentrations of insulin, NEFA, triglyceride, adiponectin, and 3-HBA were measured using insulin enzyme linked immunoassay kit (Morinaga, Yokohama, Japan), NEFA C-test (Wako Pure Chemical Industries, Tokyo), triglyceride E-test (Wako), mouse adiponectin ELISA kit (Otsuka Pharmaceuticals, Tokyo), and beta Hydroxybutyrate (beta HB) Assay Kit (Abcam, Cambridge, UK), respectively, according to the instructions provided by the manufacturer.

### Microarray analysis

Gene expression was profiled with Mouse Gene Expression 8 × 60 K Microarray (Agilent, Santa Clara, CA) by Takara Bio Inc (Tokyo). Cyanine-3 (labeled cRNA was prepared from 0.1 μg total RNA using the Low Input Quick Amp Labeling Kit (Agilent)). Fragmentation, hybridization, and washing steps were also carried out as recommended by the manufacturer (Agilent). Slides were scanned on the Agilent SureScan Microarray Scanner G2600D using one color scan setting for 8 × 60 K array slides. The scanned images were analyzed with Feature Extraction Software 11.5.1.1 (Agilent) using default parameters to obtain the intensity of the processed signal.

### Metabolomic analysis

Approximately 50 mg of frozen periovarian adipose tissue and liver was plunged into 1,500 µL of 50% acetonitrile/Milli-Q water containing internal standards (solution ID: 304-1002, Human Metabolome Technologies, Inc., Tsuruoka, Japan) at 0 °C in order to inactivate the tissue enzymes. The tissue was homogenized thrice at 1,500 rpm for 120 sec using a tissue homogenizer (Micro Smash MS100R, Tomy Digital Biology Co., Tokyo) and then the homogenate was centrifuged at 2,300 × *g* and 4 °C for 5 min. Subsequently, 800 µL of the upper aqueous layer was centrifugally filtered through a Millipore 5-kDa cutoff filter at 9,100 × *g* and 4 °C for 120 min to remove proteins. The filtrate was centrifugally concentrated and re-suspended in 50 µL of Milli-Q water for Capillary Electrophoresis-Mass Spectrometry (CE-MS) analysis. Metabolome measurements were carried out through a facility service at Human Metabolome Technologies.

### Cell cultures and differentiation

3T3-L1 adipocytes were cultured in DMEM (Nacalai Tesque, Kyoto, Japan) supplemented with 10% FBS until two days post-confluence. Then (day 0), the cells were treated with adipogenic mixture containing 0.5 mM 3-isobutyl-1-methylxanthine (Nacalai), 1 μM dexamethasone (Sigma-Aldrich, St. Louis, MO), and 1 μM insulin (Nacalai) for 48 hr. After 48 hr (day 2), medium was replaced with DMEM supplemented with 10% FBS. Until day 7, the cells were maintained in DMEM with 10% FBS.

### Effects of 3-hydroxybutyric acid on 3T3-L1 adipocytes

On day 7 after the induction of differentiation, the medium of 3T3-L1 cells was replaced with Krebs-Ringer Bicarbonate buffer (KRBB), composed of 25 mM NaHCO_3_ (Nacalai), 119 mM NaCl (Nacalai), 4.74 mM KCl (Nacalai), 1.19 mM MgCl_2_ (Nacalai), 1.19 mM KH_2_PO_4_ (Nacalai), 2.54 mM CaCl_2_ (Nacalai), 10 mM 4-(2-hydroxyethyl)-1-piperazineethanesulfonic acid (Nacalai), 0.05 mM Bovine Serum Albumin solution (Sigma), and 25 mM glucose (Otsuka), with various concentrations of 3-Hydroxybutyric acid (Sigma). The cells were collected after 24 hr, then grown at 37 °C under 5% CO_**2**_ fully humidified air environment.

### RNA extraction, cDNA synthesis, and quantitative real-time PCR

Total RNA was isolated using TRI-Reagent (Sigma) based on the method recommended by the manufacturer. The total amount of RNA was quantified by measurement of optical density (OD) at 260 nm. All RNA purity was determined by measuring the 260/280 and 260/230 ratios with Nanodrop instrument. The cDNA was synthesized using the PrimeScript RT Master Mix (Takara) according to the instructions supplied by the manufacturer. Real-time PCR was performed using THUNDERBIRD SYBR qPCR Mix (Toyobo, Osaka, Japan) on the LightCycler System (Roche, Basel, Switzerland) according to the protocol provided by the manufacturer. The sequences of primers used for real-time PCR are described in Supplementary Table 1.

### Western blot analysis

Cell lysates of 3T3-L1 cells were collected. Then, 4.3 μg of cell lysate protein was subjected to SDS-PAGE under reducing conditions, transferred, and blotted with HRP-conjugated anti-rat Adiponectin antibody (R&D Systems, Minneapolis, MN) followed by HRP-conjugated rat IgG antibody (Amersham Biosciences, Little Chalfont, UK). The membrane was stripped and reproved with anti-mouse β-actin Clone AC-15 (Sigma), followed by HRP-conjugated mouse IgG antibody (Amersham Biosciences).

### Plasmids

pGL3 basic were purchased from Promega (Madison, WI). a10390Luc and a908Luc were reported previously or constructed in our laboratory, as described in detail elsewhere^26,27^.

### Luciferase reporter assay

On day 7 after the induction of differentiation as stated above, the medium of 3T3-L1 cells in 12-well plates was changed to Opti-MEM (Invitrogen, Carlsbad, CA), and the cells were transfected with luciferase reporter plasmids using Lipofectamine 3000 Transfection Reagent (Invitrogen) according to the protocol provided by the manufacturer. Transfection was performed using 2 ng CMV-Renilla (internal standard) and 100 ng reporter plasmids. The next day (day 8), the medium was changed to Krebs-Ringer Bicarbonate buffer, composed as stated above, with or without 10 mM 3-Hydroxybutyric acid (Sigma) or 1 μM pioglitazone (Takeda, Osaka, Japan). After 24-hr incubation, luciferase reporter assays were performed using Promega Dual-Luciferase Reporter Assay System (Promega). Luciferase values were normalized by an internal CMV-Renilla control and expressed as relative luciferase activity.

### Genomic DNA purification and bisulfite sequencing analysis

DNA was purified by phenol-chloroform extraction. Cells were lysed in lysis buffer (50 mM Tris-HCl (pH 7.8), 100 mM ethylenediaminetetraacetic acid (EDTA; pH 8.0), 100 mM NaCl, 1% SDS, and 500 μg/mL proteinase K) at 55 °C overnight. Bisulfite conversion was performed using the MethylEasy Xceed (Takara) according to the instructions provided by the supplier. The converted DNA was amplified by PCR using primers as described previously^28^. PCR was performed using the TaKaRa EpiTaq HS (Takara) according to the protocol provided by the manufacturer. PCR products were cloned into bacteria using the TOPO TA Cloning Kit (Invitrogen). Eight clones for each sample were sequenced. The primer sequences that were used for PCR are listed in Supplementary Table 2.

### Chromatin immunoprecipitation assay

Chromatin immunoprecipitation (ChIP) assay was performed as described previously^45^ with β-hydroxybutyryl-Histone H3(Lys9) rabbit pAb (PTM BIOLABS, Chicago, IL), anti-acetyl-Histone H3(Lys9) pAb (Merck Millipore, Burlington, NJ), anti-Histone H3(dimethyl K9) antibody (Abcam) ChromPure Rabbit IgG (Jackson Immuno Research, Baltimore, PA), and ChromPure Rabbit IgG (Jackson Immuno Research), and the primers described in Supplementary Table 3.

### Statistical analysis

All data were expressed as mean ± SEM values. Differences between two groups were examined for statistical significance by the Student’s t-test, and among three groups by one-way analysis of variance (ANOVA) followed by Tukey-Kramer test. A *P* value < 0.05 denoted the presence of a statistically significant difference. The JMP Pro 13.1.0 software (SAS Institute. Inc., Cary, NC, USA) was used in all statistical analyses.

## Electronic supplementary material

Supplementary information
