# Supplementary material for: Metabolomic and microarray analyses of adipose tissue of dapagliflozin-treated mice, and effects of 3-hydroxybutyrate on induction of adiponectin in adipocytes
Source: Sci Rep. 2018 Jun 11;8:8805. doi: 10.1038/s41598-018-27181-y (PMC5995811; doi:10.1038/s41598-018-27181-y)
Supplement: Supplementary file 1 — Supplementary information [file 41598_2018_27181_MOESM1_ESM.pdf]

Supplementary information

Metabolomic and microarray analyses of adipose tissue of dapagliflozin-treated mice, and effects of 3-hydroxybutyrate on induction of adiponectin in adipocytes

Shigeki Nishitani[1], Atsunori Fukuhara[1, 2], Jihoon Shin[1, 3], Yosuke Okuno[1], Michio Otsuki[1] and Iichiro Shimomura[1]

[1]Department of Metabolic Medicine, Osaka University Graduate School of Medicine, 2-2, Yamadaoka, Suita, Osaka, Japan

[2]Department of Adipose Management, Osaka University Graduate School of Medicine

[3]Department of Diabetes Care Medicine, Osaka University Graduate School of Medicine

Corresponding author:

Atsunori Fukuhara, MD

Department of Adipose Management, Osaka University Graduate School of Medicine, Osaka 565-0871, Japan

Tel: +81-6-6879-3732, Fax: +81-6-6879-3739

E-mail: fukuhara@endmet.med.osaka-u.ac.jp

Supplementary Table 1 Primers used for quantitative real-time PCR

Gene		Sequence (5’ to 3’)
Adiponectin	Forward	GTTCTACTGCAACATTCCGG
	Reverse	TACACCTGGAGCCAGACTTG
IL-6	Forward	ACAACCACGGCCTTCCCTACTT
	Reverse	CACGATTTCCCAGAGAACATGTG
MCP-1	Forward	CCACTCACCTGCTGCTACTCAT
	Reverse	TGGTGATCCTCTTGTAGCTCTCC
PAI-1	Forward	TCAGCCCTTGCTTGCCTCAT
	Reverse	GCATAGCCAGCACCGAGGA
PPAR-γ	Forward	ATCTTAACTGCCGGATCCACAA
	Reverse	GCCCAAACCTGATGGCATT
TNF-α	Forward	CCATTCCTGAGTTCTGCAAAG
	Reverse	GCAAATATAAATAGAGGGGGGC
36B4	Forward	GCTCCAAGCAGATGCAGCA
	Reverse	CCGGATGTGAGGCAGCAG

IL-6, interleukin 6; MCP-1, Monocyte chemoattractant protein-1; PAI-1, plasminogen activator inhibitor 1; PPAR-γ, peroxisome proliferative activated receptor gamma; TNF-α, tumor necrosis factor alpha; 36B4, ribosomal protein, large, P0 (Rplp0)

Supplementary Table 2 Primer sequences used for bisulfite sequencing analysis

Gene		Sequence (5' to 3')
Mouse Adiponectin -1,168 ~ -1,144	Forward	TGGAGGAAGTAGATGTTTGGTTAGT
	Reverse	CAAAACAATACCTTAAAAACCTATC

Supplementary Table 3 Primers used for ChIP-pPCR analysis

Region on the adiponectin gene		Sequence (5’ to 3’)
-700	-705 ~ -684	ACCCCTGAACTTGCTTCACACC
	-628 ~ -606	TGCTAGACCGGATCCCATACTGA
-500	-549 ~ -528	TGCATGCATATTTGCACACCAA
	-502 ~ -481	TCAATTCCCAGCACCCACAGTA
-300	-371 ~ -350	ATGGCTGAACCACACAGCTTCA
	-274 ~ -253	AGGGGTCAGGAGACCTCCCTTT
-100	-109 ~ -88	TTCCCAGACCCAAGCTGGATTA
	-29 ~ -8	CAACCCAGTCAAGGCCAATAGC
100	48 ~ 68	GGCCACTTTCTCCTCATTTCT
	84 ~ 104	TTTGGTGTCGTCAGATCCACT
300	226 ~ 247	GGAAACATGGTTGGGGCAGATA
	342 ~ 362	GGCATCCTGCCATCAAATGAA
2,300	2,251 ~ 2,271	TGATTGGGTTGTGCCATTGTG
	2,348 ~ 2,369	GGCATTTGCCCAATGTGTATGA
4,500	4,490 ~ 4,510	TCCCCATGGAAAAGATTGGTG
	4,597 ~ 4,618	CCGCCATTGCTCTGAGACTTTT
7,300	7,200 ~ 7,220	TGCCTCAAAAACCCAAGAGGA
	7,295 ~ 7,315	TGTTTTCAGGCAGGCAGCTTT
8,800	8,741 ~ 8,734	TTCCTCTTAATCCTGCCCAGT
	8,798 ~ 8,817	ATCCAACCTGCACAAGTTCC
9,700	9,645 ~ 9,666	TAGGTATCTGCCCAGCTCTTGT
	9,730 ~ 9,750	GGTTTCTACCCCACCTTTGAT

Supplementary Methods

Measurements of plasma corticosterone and leptin

Plasma concentrations of corticosterone and leptin were measured using Corticosterone Enzyme Immunoassay Kit (Arbor Assays, Michigan, USA) and leptin enzyme linked immunoassay kit (Morinaga), respectively, according to the instructions provided by the manufacturer.

Assay of pyruvate carboxylase activities

Pyruvate carboxylase activities were measured as described by Crabtree [1], with some modifications. Briefly, adipose tissues were sonicated in 100 mM Tris/HCl buffer, pH 8.0, and corrected by protein concentrations. The wells in 96 well plate contained in a final volume of 200μL: 90 mM Tris/HCl buffer, 50 mM, $NaHCO_3$, 5 mM $MgCl_2$, 100 mM acetyl-CoA, 0.25 mM 5,5'-dithiobis-(2-nitrobenzoic acid), 10 U citrate synthase, 5 mM ATP, 10 mM pyruvate, and 50 μL of adipose tissue extract. The rate of increase in OD412 was measured. Control samples without pyruvate were used to correct for the increase in OD412 caused by hydrolysis of acetyl-CoA in the presence of adipose tissue extract.

Assay of Succinyl-CoA:3-ketoacid CoA-transferase (SCOT) activities

SCOT activity was measured in the direction of succinyl-CoA formation, as described by Sakazaki [2], with some modifications. Briefly, adipose tissues were sonicated in 50 mM sodium phosphate, pH 8.0, 0.1% Triton X-100, and corrected by protein concentrations. The wells in 96 well plate contained, in a final volume of 200μL: 50 mM Tris-HCl buffer, pH 8.5, 10 mM $MgCl_2$, 4 mM iodoacetamide, 30 μmol/L acetoacetyl-CoA, and 10 μL of adipose tissue extract. After measurement of OD303, 10 μmol of sodium succinate was added, and sodium succinate-dependent decreased of OD303 was measured.

Effects of 3-hydroxybutyric acid on glycerol release from 3T3-L1 adipocytes

On day 7 after differentiation, the medium of 3T3-L1 cells were replaced with Krebs-Ringer Bicarbonate buffer (KRBB; composition was stated in Methods section) supplemented with 0 (control) or 3 mM 3-HBA, or DMEM (Nacalai) supplemented with 1 μM isoproterenol (Sigma) and incubated for 6 hours. After 0, 1, 3, or 6 hours of incubation, supernatant of differentiated 3T3-L1 cells were collected respectively. 2.5 μL of each supernatant samples and 200 μL of Free Glycerol Reagent (Sigma) were added into wells in 96 well plate. After incubation 37°C for 5 minutes, glycerol levels were quantified by measurement of OD540.

References

1 Crabtree, B., Newsholme, E. A. & Higgins, S. J. Activities of Pyruvate Carboxylase, Phosphoenolpyruvate Carboxylase and Fructose Diphosphatase in Muscles from Vertebrates and Invertebrates. *Biochem. J.* **130**, 391-+, doi:Doi 10.1042/Bj1300391 (1972).

2 Hasan, N. M. *et al.* Lower succinyl-CoA:3-ketoacid-CoA transferase (SCOT) and ATP citrate lyase in pancreatic islets of a rat model of type 2 diabetes: knockdown of SCOT inhibits insulin release in rat insulinoma cells. *Arch. Biochem. Biophys.* **499**, 62-68, doi:10.1016/j.abb.2010.05.007 (2010).

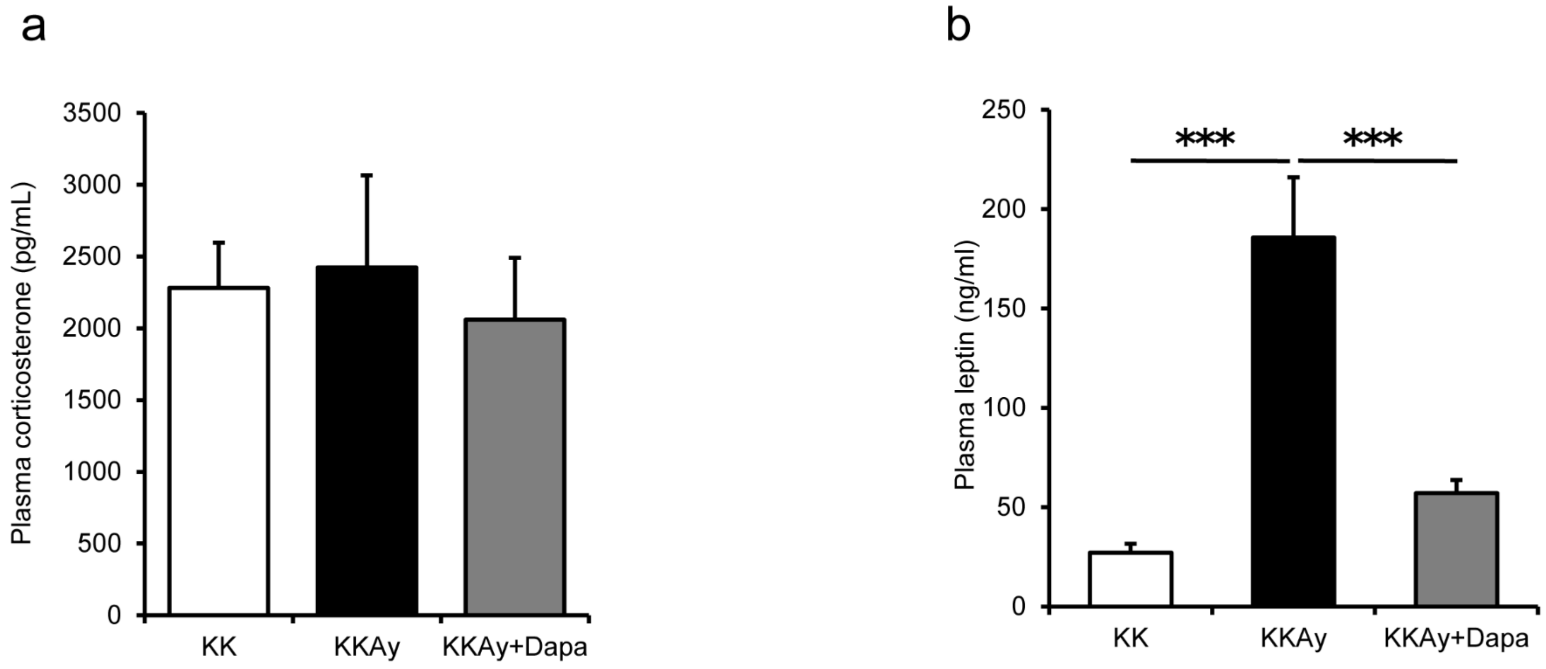

Supplementary Figure S1. Plasma levels of corticosterone and leptin. (a) Plasma corticosterone, and (b) plasma leptin levels were measured after 5 weeks of treatment with dapagliflozin. Data are mean ± SEM (n=6). ***p<0.001, by one-way ANOVA followed by post hoc analysis (Tukey-Kramer test).

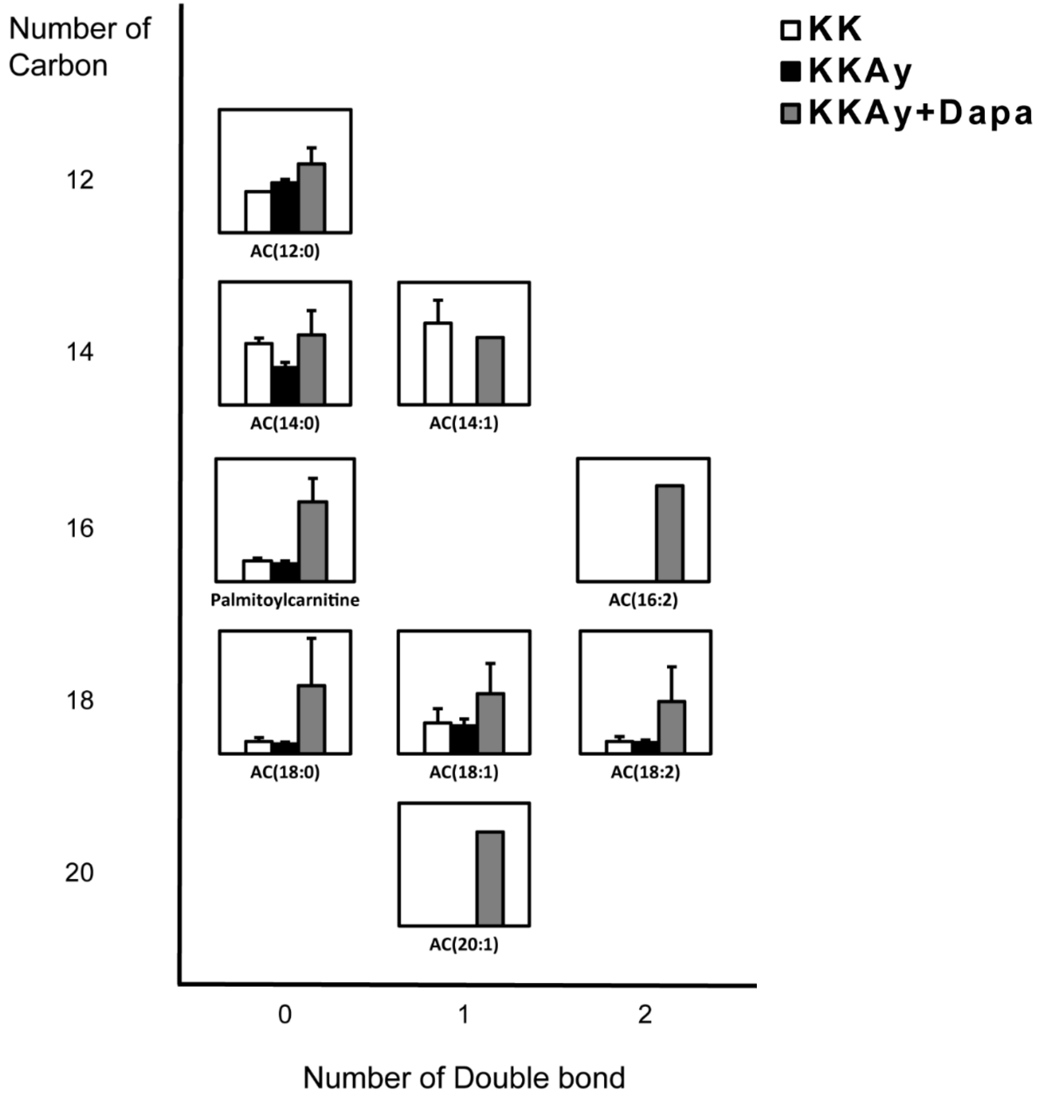

Supplementary Figure S2. Metabolomic analyses of acylcarnitine in periovarian WAT (ovaWAT). Relative levels of acylcarnitine after 5 weeks of dapagliflozin treatment. Data are mean ± SEM (n=4).

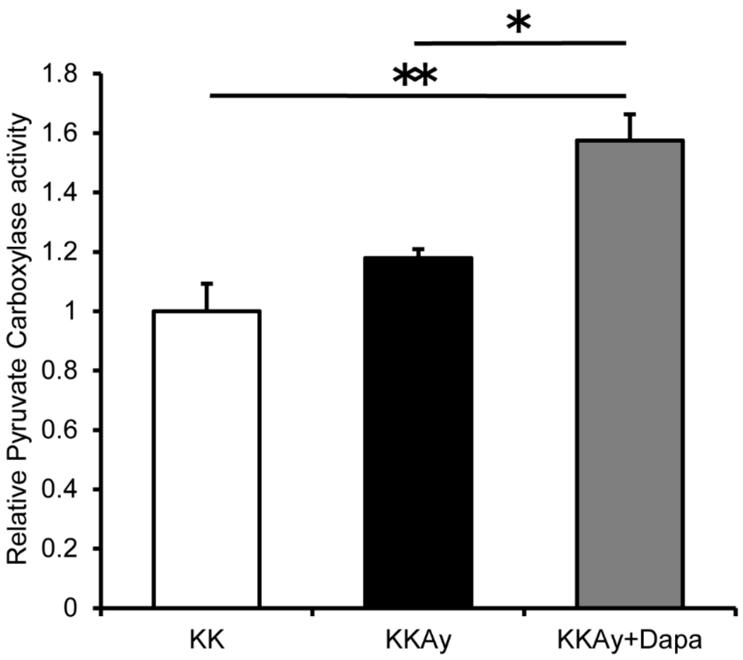

Supplementary Figure S3. Relative Pyruvate Carboxylase activity of ovaWAT. Enzyme activities were measured after 5 weeks of dapagliflozin treatment. Data are normalized to the values of activities of KK mice, and expressed as mean ± SEM (n=4). *p<0.05, **p<0.01, by one-way ANOVA followed by post hoc analysis (Tukey-Kramer test). Additional details are given in the Supplementary Methods section.

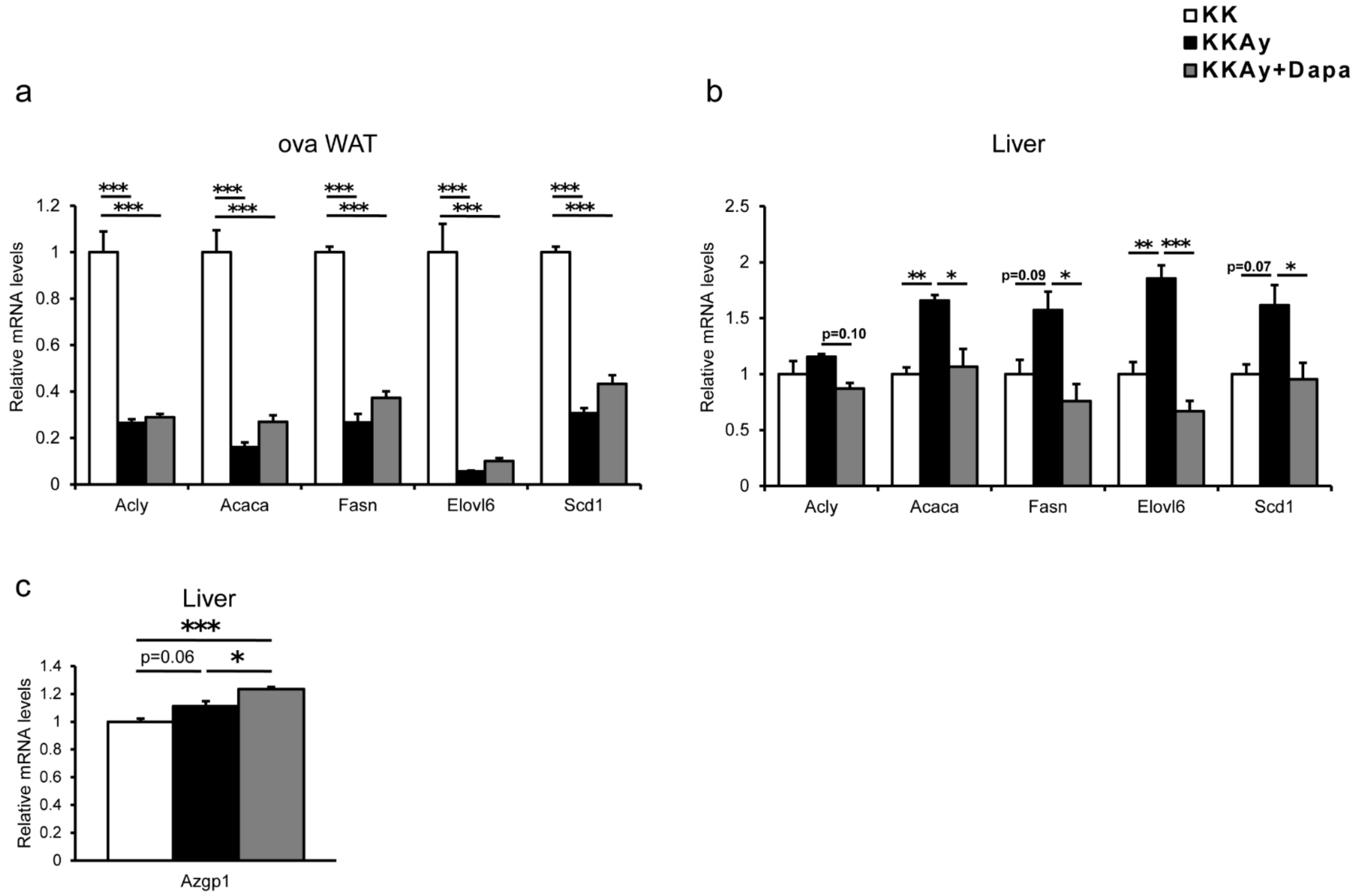

Supplementary Figure S4. Microarray analyses of gene expressions associated with lipogenesis. Relative expression levels of genes associated with lipogenesis in ovaWAT (a) and liver (b), and Azgp1 mRNA expression levels in liver after 5 weeks of dapagliflozin treatment. Data are normalized to the values of gene expression levels of KK mice, and expressed as mean ± SEM (n=4). *p<0.05, **p<0.01, ***p<0.001, by one-way ANOVA followed by post hoc analysis (Tukey-Kramer test). Acyl, adenosine triphosphate citrate lyase; Acaca, acetyl-Coenzyme A carboxylase alpha; Fasn, fatty acid synthase; Elovl6, elongation of very long chain fatty acids protein 6; Scd1, stearoyl-Coenzyme A desaturase 1.

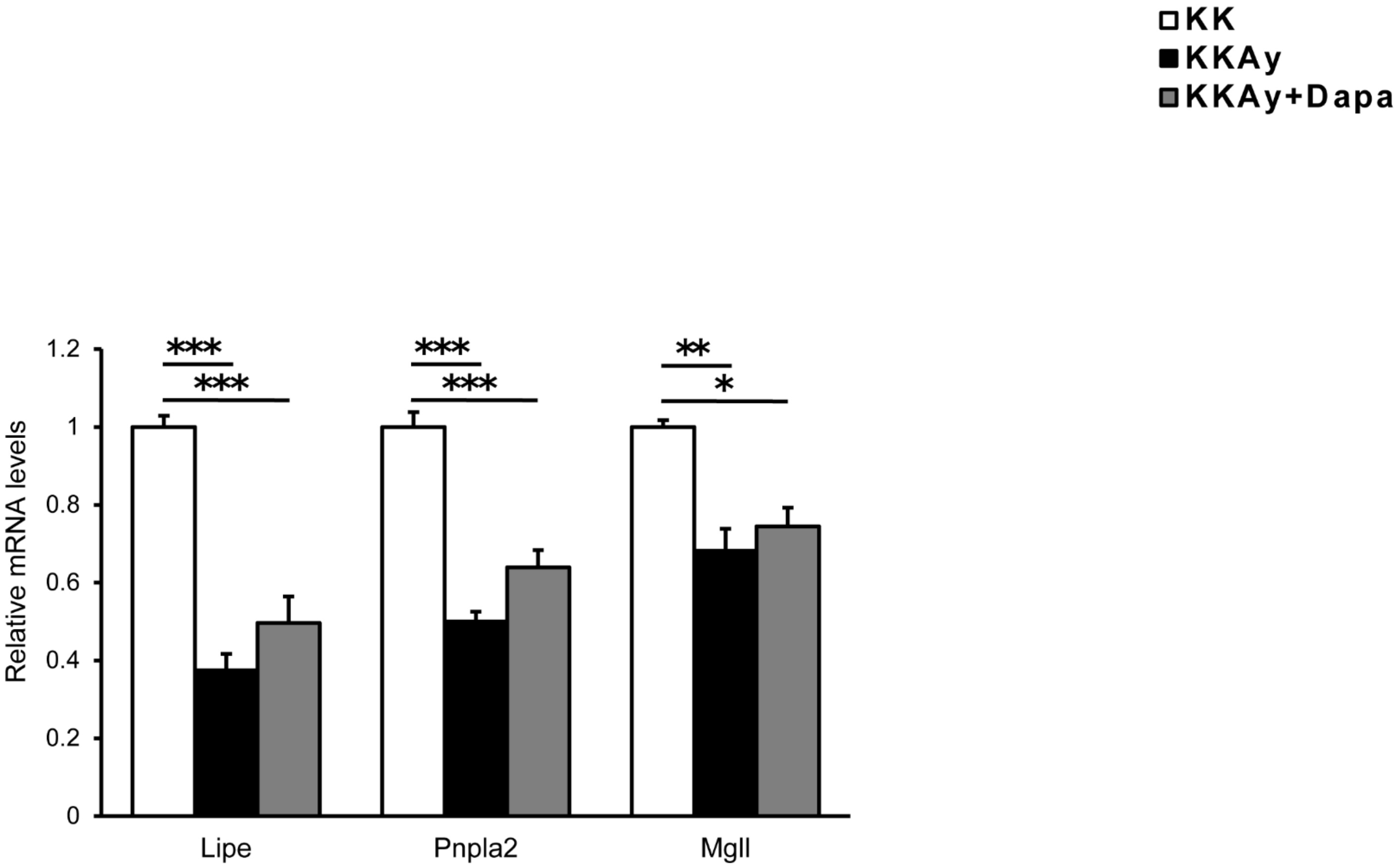

Supplementary Figure S5. Microarray analyses of gene expressions associated with lipolysis in ovaWAT after 5 weeks of dapagliflozin treatment. Data are normalized to the values of gene expression levels of KK mice, and expressed as mean ± SEM (n=4). *p<0.05, **p<0.01, ***p<0.001, by one-way ANOVA followed by post hoc analysis (Tukey-Kramer test). Lipe, hormone sensitive lipase; Pnpla2, patatin-like phospholipase domain containing 2; Mgll, monoglyceride lipase.

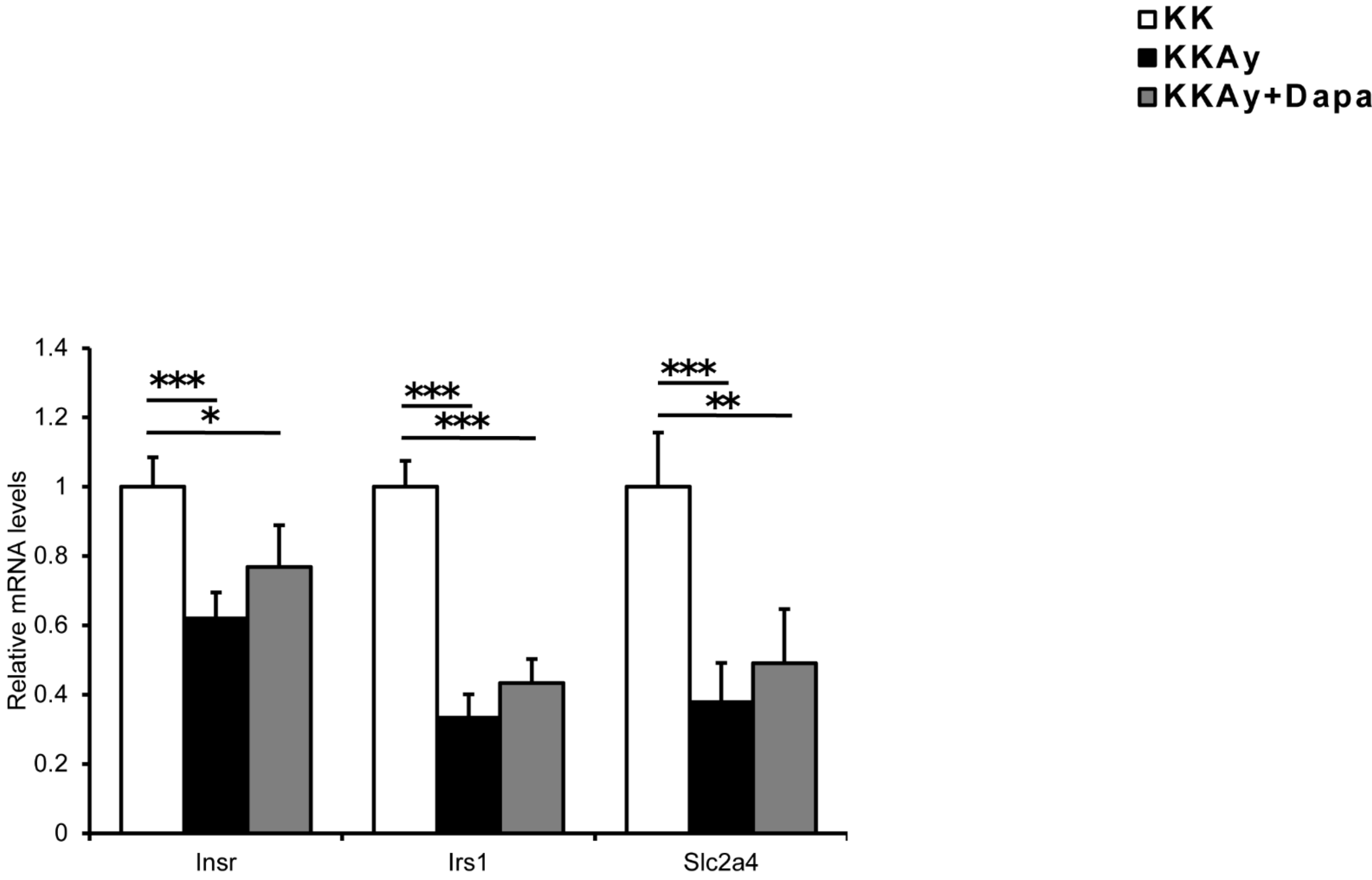

Supplementary Figure S6. Microarray analyses of gene expressions associated with insulin signaling in ovaWAT after 5 weeks of dapagliflozin treatment. Data are normalized to the values of gene expression levels of KK mice, and expressed as mean ± SEM (n=4). *p<0.05, **p<0.01, ***p<0.001, by one-way ANOVA followed by post hoc analysis (Tukey-Kramer test). Insr, insulin receptor; Irs1, insulin receptor substrate 1; Slc2a4, solute carrier family 2, facilitated glucose transporter member 4.

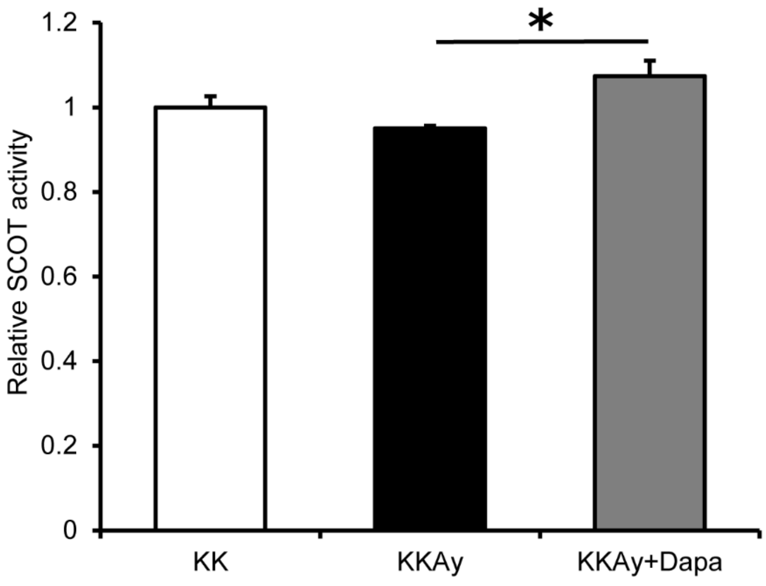

Supplementary Figure S7. Relative succinyl-CoA-3-oxaloacid CoA transferase (SCOT) activity of ovaWAT. Enzyme activities were measured after 5 weeks of dapagliflozin treatment. Data are normalized to the values of activities of KK mice, and expressed as mean ± SEM (n=4). *p<0.05, **p<0.01, by one-way ANOVA followed by post hoc analysis (Tukey-Kramer test). Additional details are given in the Supplementary Methods section.

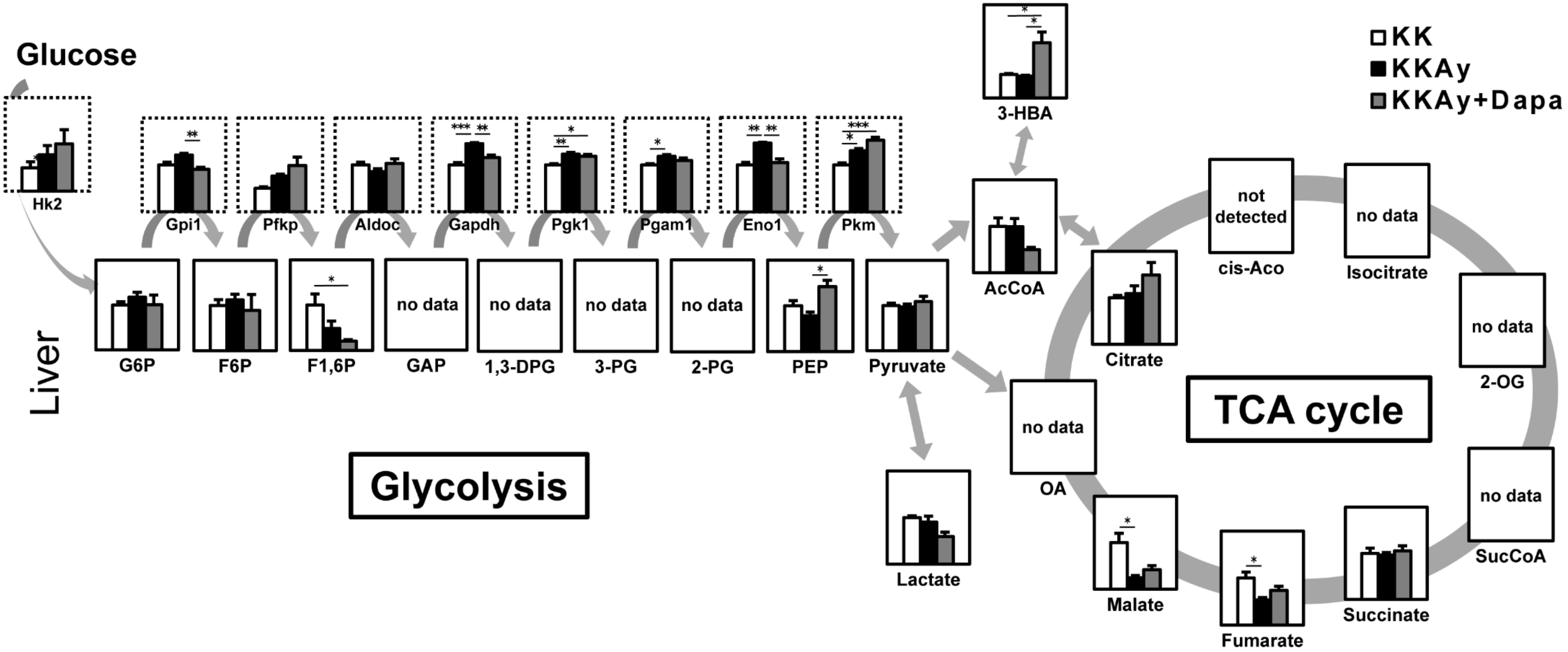

Supplementary Figure S8. Results of metabolomic and microarray analyses of liver after 5 weeks of dapagliflozin treatment. Relative levels of metabolites and gene expressions associated with glycolytic and tricarboxylic cycle (TCA cycle) pathways. Relative levels of metabolites and gene expressions were surrounded by solid line and bottled line, respectively. Data are normalized to the values of metabolites or gene expression levels of KK mice, and expressed as mean ± SEM (n=4). *$p<0.05$, **$p<0.01$, ***$p<0.001$, by one-way ANOVA followed by post hoc analysis (Tukey-Kramer test).

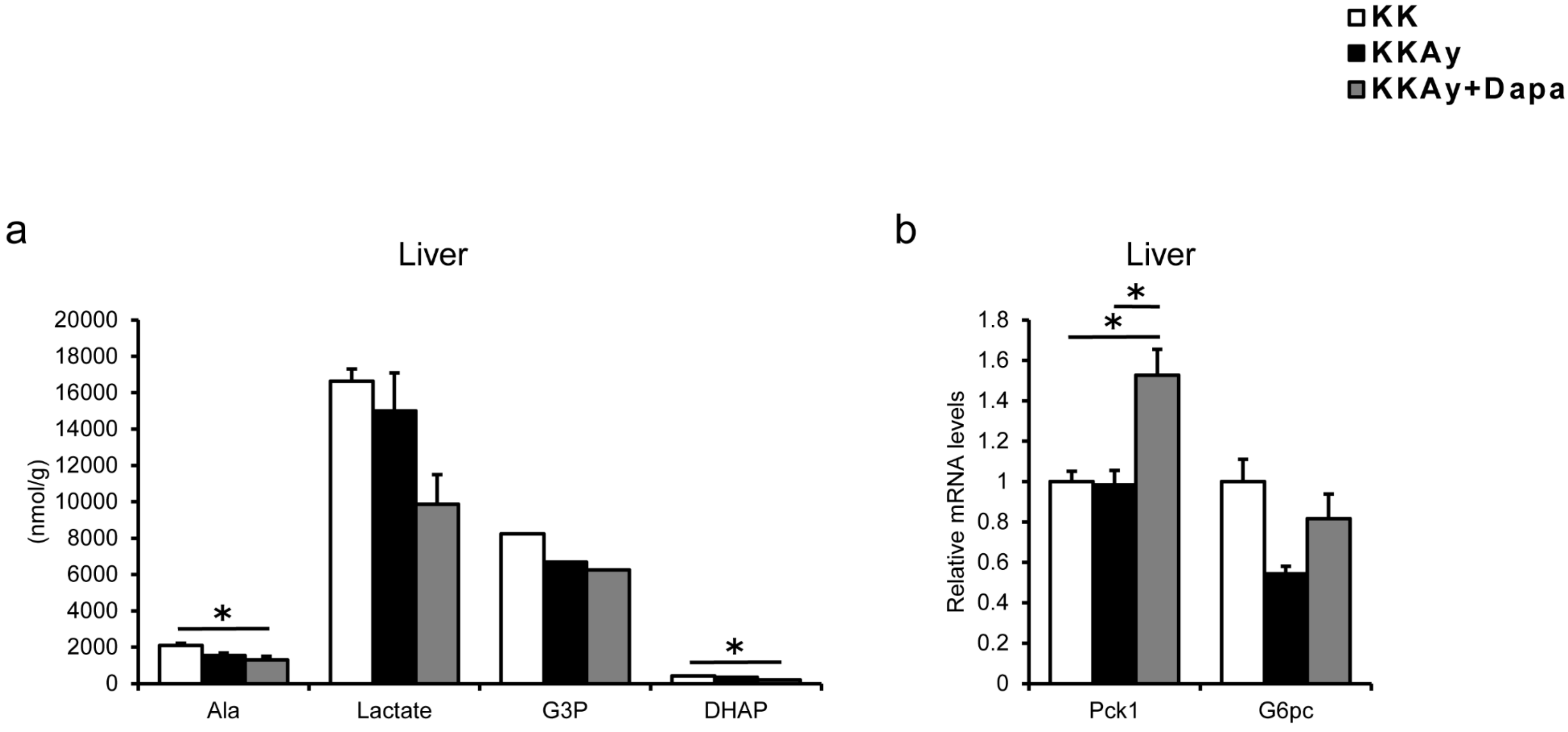

Supplementary Figure S9. Results of metabolomic and microarray analyses of liver associated with gluconeogenesis after 5 weeks of dapagliflozin treatment. Quantitative analysis of metabolites (a) and relative gene expression levels (b) associated with gluconeogenesis. Gene expression levels are normalized to the values of the levels of KK mice. Data are mean ± SEM (n=4). *$p<0.05$, by one-way ANOVA followed by post hoc analysis (Tukey-Kramer test). Ala, alanine; G3P, glycerol 3-phosphate; DHAP, dihydroxyacetone phosphate; Pck1, phosphoenolpyruvate carboxykinase 1, cytosolic; G6pc, glucose-6-phosphatase, catalytic subunit.

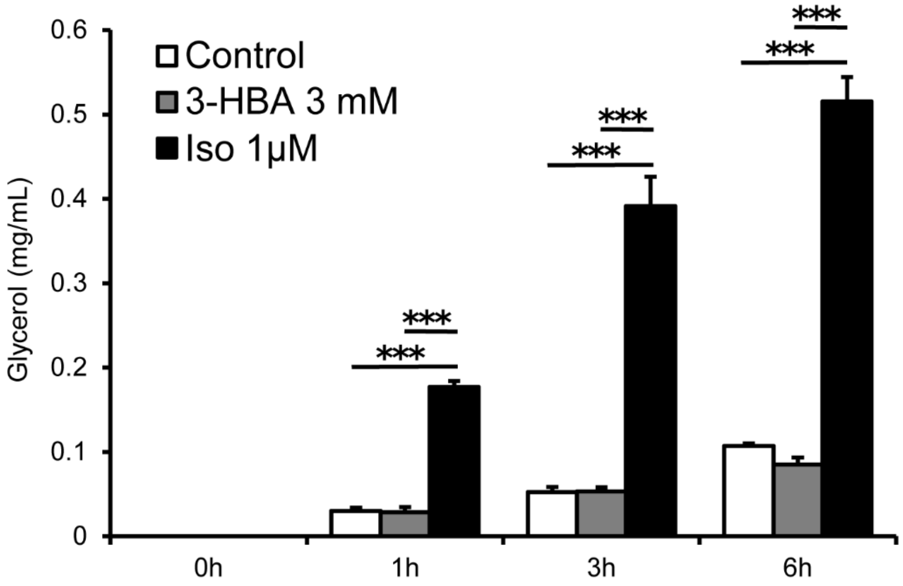

Supplementary Figure S10. Effects of 3-HBA on glycerol release from 3T3-L1 adipocytes. On day 7 after differentiation, the medium of 3T3-L1 cells were replaced with KRBB supplemented with 0 (control) or 3 mM 3-HBA, or DMEM supplemented with 1 μM isoproterenol and incubated for 6 hours. After 0 hour, 1 hour, 3 hours, and 6 hours incubating, supernatant of differentiated 3T3-L1 cells were collected respectively, and glycerol levels of supernatant were measured. Data are mean ± SEM (n=3). ***$p<0.001$, by one-way ANOVA followed by post hoc analysis (Tukey-Kramer test).

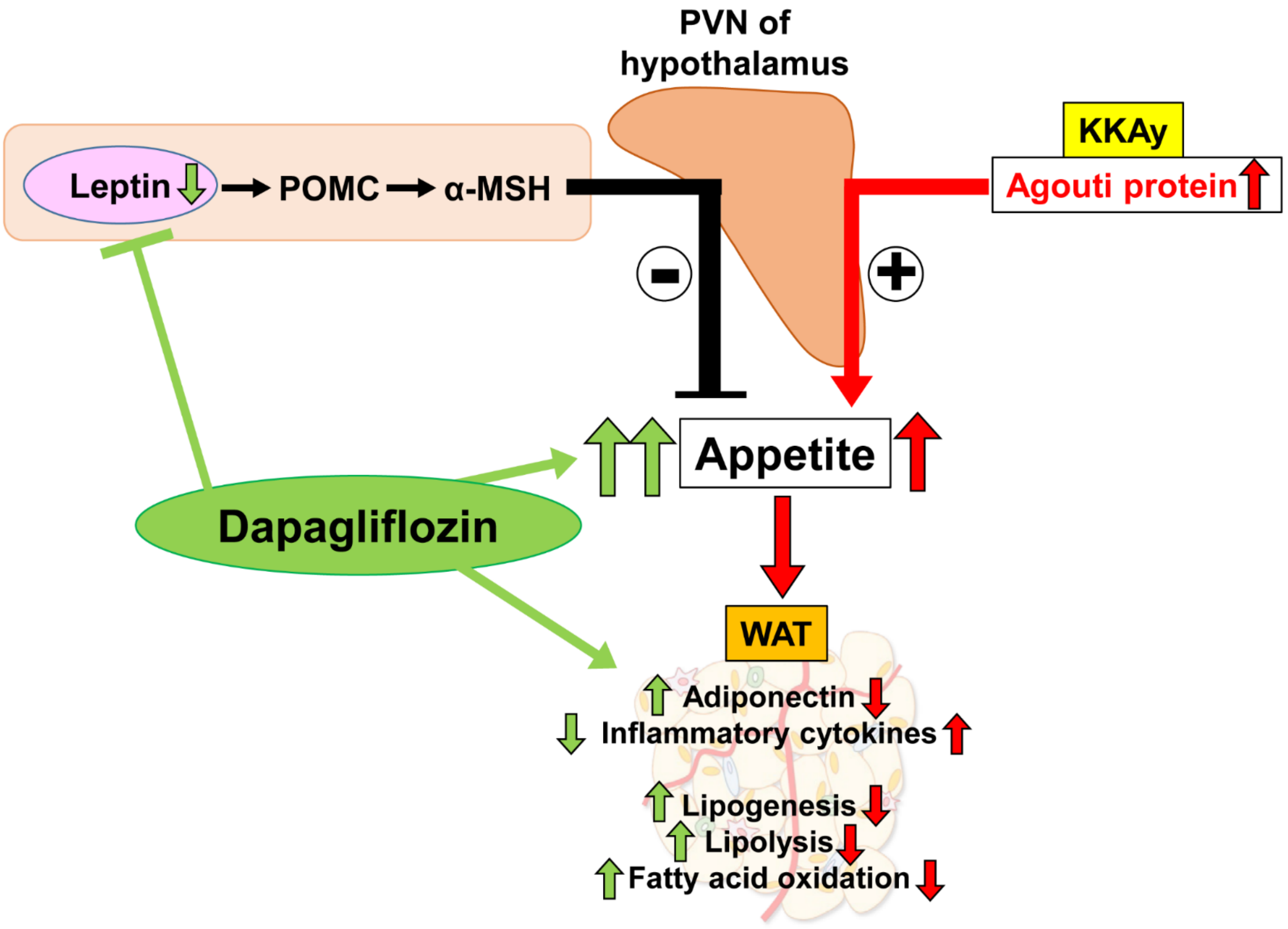

Supplementary Figure S11. Schematic diagram of the effects of Agouti and dapagliflozin. Ectopic expression of agouti protein in KKAy mice stimulates appetite (red arrow). Excess calorie intake due to hyperphagia results in reduction of adiponectin expression, induction of inflammatory cytokines, and inhibition of lipogenesis, lipolysis, and fatty acid oxidations in WAT (red arrows). Treatment with dapagliflozin reduces plasma leptin levels (green arrow), enhances further hyperphagia (green arrow), whereas, it improves dysregulation of adipocytokines and lipid metabolism in WAT (green arrows). POMC, Pro-opiomelanocortin); α-MSH, alpha-melanocyte-stimulating hormone; PVN, paraventricular nucleus.

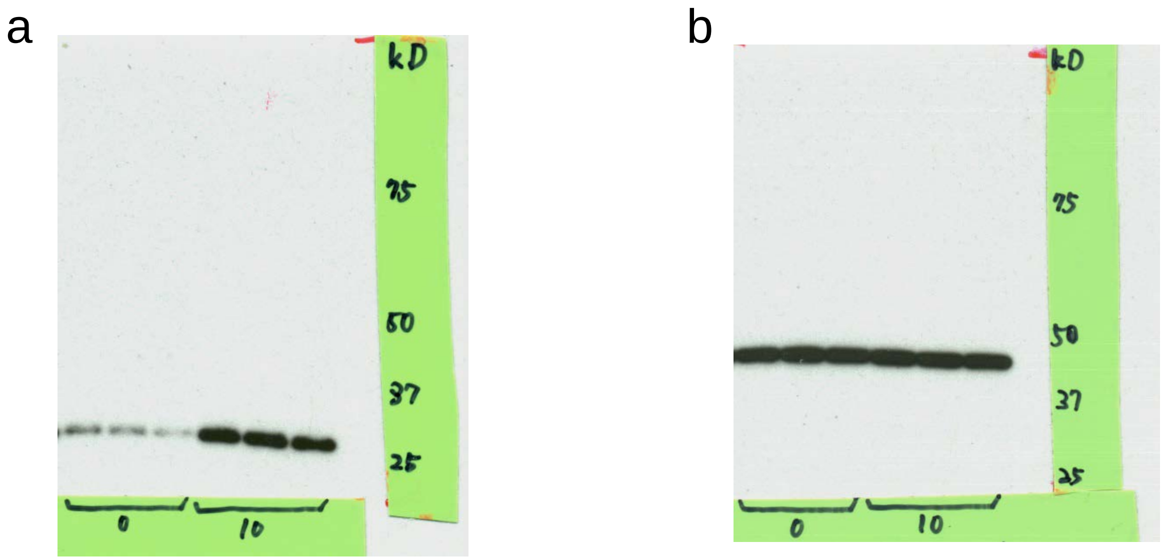

Supplementary Figure S12. Full-length western blot images for 3T3-L1 adipocytes treated with or without 3-HBA associated with Figure 4g. On day 7 after differentiation, the medium of 3T3-L1 cells were replaced with KRBB supplemented with 0 or 10 mM 3-HBA and incubated for 24 hours. After 24 hours incubation, cells were collected respectively, and western blot analyses were performed as stated in methods section. (a) adiponectin, (b) β-Actin.
